# Evaluation of the Regenerative Potential of Platelet-Lysate and Platelet-Poor Plasma Derived from the Cord Blood Units in Corneal Wound Healing Applications: An In Vitro Comparative Study on Corneal Epithelial Cells

**DOI:** 10.3390/cimb44100303

**Published:** 2022-09-22

**Authors:** Panagiotis Mallis, Efstathios Michalopoulos, Eirini Faidra Sarri, Elena Papadopoulou, Vasiliki Theodoropoulou, Michalis Katsimpoulas, Catherine Stavropoulos-Giokas

**Affiliations:** 1 Hellenic Cord Blood Bank (HCBB), Biomedical Research Foundation Academy of Athens, 4 Soranou Ephessiou, 115 27 Athens, Greece; 2 Experimental Surgery Unit, Center of Clinical, Experimental Surgery and Translational Research, Βιοmedical Research Foundation of the Academy of Athens, 4 Soranou Ephessiou, 115 27 Athens, Greece

**Keywords:** cord blood, platelet lysate, platelet poor plasma, growth factors, ophthalmology, corneal epithelial cells, corneal wound healing

## Abstract

Background: Cord blood platelet lysate (CB-PL) and cord blood platelet poor plasma (CB-PPP) have been applied with success in wound healing applications. Pathologies such as Sjogrens’s Syndrome (SS) and chronic graft versus host disease (cGVHD) can lead to severe ophthalmology issues. The application of CB-PL and CB-PPP may be strongly considered for damaged cornea healing. This study aimed to the evaluation of the beneficial properties of CB-PL and CB-PPP in corneal wound healing applications. Methods: Initially, the CB-PL and CB-PPP were produced from donated cord blood units (CBUs), followed by biochemical analysis. Corneal epithelial cells (CECs) were isolated from wistar rats and then cultured with medium containing 20% *v*/*v* either of CB-PL or CB-PPP. To define the impact of CB-PL and CB-PPP, biochemical, morphological analysis, scratch-wound assays, and immunoassays in CECs were performed. Results: CB-PL and CB-PPP were characterized by good biochemical parameters, regarding their quality characteristics and biomolecule content. CECs’ morphological features did not change after their cultivation with CB-PL or CB-PPP. A scratch wound assay and molecular analysis of CECs expanded with CB-PL indicated higher migratory capacity compared to those cultured with CB-PPP. Conclusion: CB-PL and CB-PPP exhibited good properties with respect to cell migration and proliferation, and could be considered an alternative source for eye drop production, to possibly be used in cornea wound healing applications.

## 1. Introduction

Dry eye syndrome (DES) is a multifactorial disease that eventually can damage the corneal epithelium, conjunctiva, accessory lacrimal, and meibomian glands, accompanied by several unfavorable events for the patients [1]. DES is one of the most common under-recognized disorders of the people aged >50 years old, and is related to variable degrees of discomfort, visual disturbance, etc., thus affecting the patients’ quality of life [1]. It has been reported in the literature that females have a greater likelihood of DES occurrence than males [2,3]. This difference in disease occurrence can be explained by the fact that an association of androgen levels with the dysfunction of the Meibomian gland has been reported [3,4,5,6,7,8,9,10,11]. Another study conducted by Schaumberg et al. [4] estimated that DES will affect more than 2.79 million people by 2030 in the USA. DES is associated with great socio-economic impact due to the increased number of hospitalizations, surgeries, and high medical costs [5,6]. It is estimated that the annual cost for typical ophthalmologist care in Europe may exceed 270 euros, while different charges exist between different countries [5,6].

DES pathology can further cause osmolality changes with deleterious issues on the ocular surface. Tear hyperosmolarity functions as an inflammatory mediator, affecting the function of the CECs [3,4,5,6,7,8,9,10,11]. Indeed, CECs loss their adhesion to the tear film, meanwhile other events such as DNA damage and apoptosis may result in further damage to the ocular surface [3,4,5,6,7,8,9,10,11]. This pathogenetic mechanism includes several inflammatory events such as the elevated levels of matrix-metalloproteinase-9 (MMP-9), tumor necrosis factor-alpha (TNF-α), interleukin (IL)-1β, Il-6, IL-17, IL-22, interferon-γ (IFN-γ), chemokine (C-C motif) ligand 2 (CCL2), and others [11]. These in turn result in several unfavorable events for the patients, such as eye dryness, pain, burning sensations, eye fatigue, redness, and blurred vision, significantly affecting the daily routine and quality of life of patients [12,13,14,15].

Current therapeutic strategies involve the use of different artificial tears, lubricants, and immunosuppressive eye drops to maintain the function of the ocular surface [6,7,8,9,16]. However, all these treatments are limited to lipids, salts, biomolecules, immunoglobulins and growth factors, that can further contribute to ocular surface regeneration [3]. Also, treatments using manufactured eye drops may contain compounds that can be proven potentially toxic and highly allergic for several patients.

To address this issue, the production of eye drops utilizing the blood derivatives such as the autologous serum has been proposed [17]. Allogeneic serum can resemble the natural composition of the tears, however, the serum has impaired regenerative potential, due to the low amount of growth factors [18]. Conversely, it has been proven that blood derivatives, such as PL, are rich in biomolecules, and thus can be potentially used as eye drops [18,19]. Indeed, platelets’ α-granules contain a significant amount of healing mediators and trophic factors, such as the platelet-derived growth factor (PDGF), transforming growth factor (TGF), fibroblast growth factor (FGF), hepatocyte growth factor (HGF), vascular endothelial growth factor (VEGF), etc. [18,19]. It is known that the interplay between PDGF, FGF, TGF, and VEGF can promote the proliferation of cells and angiogenesis, resulting in advanced regeneration of the damaged tissue [20,21,22]. Currently, CB derivatives, such as the platelet rich plasma (PRP) and PL, have been applied with great success in several approaches involving skin lesions, occurring due to infection of Epidermolysis bullosa and also in patients suffering from diabetic ulcers [23,24,25,26,27,28]. Additionally, it has been reported that the CB-PPP contains a significant amount of growth factors, which may also exert wound healing properties [23]. Therefore, the CB-PPP may be used for the effective care of different type of wounds. However, a comprehensive investigation of its properties is required to acquire safe conclusions.

Under these circumstances, CB-PL and CB-PPP serving as a tool for wound and damaged cornea healing could be considered. For this purpose, the cord blood units (CBUs) that are donated in FaCT-Accredited public cord blood banks (CBBs), can serve as an additional source to produce both derivatives. CBUs are representing a valuable source of CD34+ stem cells, which are mostly applied in patients suffering from hematological malignancies, as an alternative approach to classical bone marrow transplantation [29,30]. For a typical CD34+ transplantation, a CBU must contain more than 1.5 × 10^9^ total nucleated cells (TNCs) and more than 2 × 10^6^ CD34+ cells [31,32,33]. Therefore, over 80% of the delivered CBUs to public CBBs are rejected [33,34]. Because of this, the non-processed CBUs will not be discarded and will potentially used to produce CB-PL and CB-PPP.

Then, these derivatives can be potentially applied as eye drops in patients suffering from DES.

In the context of using CB-PL and CB-PPP as eye drops, limited evidence of their impact on corneal wound healing applications has been provided in the literature. The aim of this study was primarily to evaluate the CB-PL and CB-PPP quality properties, and secondarily, to investigate the potential of using both CB-derivatives in corneal wound healing applications, performing in vitro experimental procedures. The results of these experiments may shed light and decipher the specific signaling pathways, implicated in cell migration, proliferation, and tissue remodeling. This article shares preliminary in vitro results for the possible use of CB-PL and CB-PPP in the corneal healing process, thus serving as allogeneic off-the-shelf eye drops.

## 2. Materials and Methods

### 2.1. Selection of Cord Blood Units

CBUs that were delivered to HCBB were used to produce CB-PL and CB-PPP, used in this study. The collection of all CBUs was performed by experienced midwives of the maternity site and obtained from end term (38–40 weeks) normal and cesarean deliveries. Informed consent regarding the donation of CBUs was signed by the mothers several hours before the delivery. CBUs collections were performed according to the standards of the Greek National Ethical Committee, and the entire study was approved by our Institution’s ethical board (Reference No. 1121, 10 June 2021). All CBUs used for the blood derivatives preparations did not meet the criteria for processing, cryopreservation, and release for administration outlined by the HCBB and FACT-NetCord accreditation (Table S1). After the collection and delivery to the HCBB, the CBUs were processed immediately. Initially, the CBUs were counted in automated hematological analyser (Sysmex XS1000i, Sysmex Europe, Norderstedt, Germany), for the determination of white blood cells (WBCs), red blood cells (RBCs), platelets (PLTs), hematocrit (HCT), and hemoglobin (HgB). CBUs that had PLT count >150 × 10^3^/μL (PLT: > 13,000 × 10^6^) and WBCs < 12 × 10^3^/μL (WBCs: < 1200 × 10^6^), were selected for the CB-PL and CB-PPP production.

### 2.2. Cord Blood Units Testing and Eligibility

All CBUs (*n* = 25) in this study were tested for active transmissible infections, e.g., HIV I/II, HCV, HAV, HBV, HCV, HTLV I/II, WNV, T.cruzi and CMV (IgM), using serological and NAT methods. Furthermore, CBUs were tested for aerobic, anaerobic bacteria and fungi using the BacT alert system. The whole procedure of CBUs processing for CB-PL and CB-PPP preparations was performed in Good Manufacturing Practices (GMP) rooms at the HCBB.

### 2.3. Cord Blood Platelet Lysate and Cord Blood Platelet Poor Plasma Production

In this study, validation of the production process of CB-PL and CB-PPP was performed. For this purpose, *n* = 25 CBUs were used, following the procedure to produce CB-PL (*n* = 25) and CB-PPP (*n* = 25). To achieve a reliable and reproducible manufacturing process, CB-PL and CB-PPP were produced and characterized following the guidelines of the Joint United Kingdom (UK) Blood Transfusion Services Professional Advisory Committee (JPAC, https://www.transfusionguidelines.org, accessed on 10 June 2022).

After the initial selection, the CBUs were centrifuged at 865 g for 15 min (acceleration 9, brake 0) at room temperature (RT). Then, isolation of the upper plasma fraction, corresponded to platelet-rich plasma (PRP), was performed. Then, a 0.5 mL PRP sample was received and the PLTs were determined using the automatic haematological analyzer. The PRP (*n* = 25) fractions obtained from all CBUs were re-centrifuged at 2500 g for 15 min (acceleration 9, brake 3), to obtain the final platelet concentrate (PC) product. Volume determination of the PC was performed using the following equation:

(1) $$\mathrm{PC}\;\mathrm{Volume}\;\left(\mathrm{ml}\right)=\frac{\left[\mathrm{PRP}\;\mathrm{PLTs}\right]\;\times \;0.01}{10^{3}/\mu L}+5$$

where PRP PLTs correspond to the PLT concentration (10^3^/μL) obtained from the PRP sample (after the first centrifugation process).

Following the above equation, the excess plasma volume was removed, which represented the PPP fraction. PPP samples (*n* = 25) were further used in this study. Then, a 0.5 mL of sample was received from the PPP and PC samples, to determine the PLT’s concentration and absolute number. Also, the determination of WBCs, RBCs, and HgB concentration was performed in all PPP and PC final products. Only PCs with PLT > 800 × 10^3^/μL (>4000 × 10^6^), WBCs < 4 × 10^3^/ μL, RBCs < 0.1 × 10^9^/μL, and HgB < 0.1 g/dL were further utilized for the next series of the experimental procedures. Finally, the PCs were stored at −80 °C for at least 48 h. To obtain the CB-PL, PCs were rapidly thawed in a water bath at 37 °C, and filtered through 0.22 μm (Sigma-Aldrich, Darmstadt, Germany), to completely remove cell remnants.

### 2.4. Cord Blood Platelet Lysate and Cord Blood Platelet Poor Plasma Quality Characterization

The evaluation of the quality characteristics of CB-PL (*n* = 25) and CB-PPP (*n* = 25) involved the determination of biochemical profile and biomolecule content. Regarding the biochemical profile, pH, glucose, lactate, APTT, PT, and fibrinogen were estimated using the blood gas analyzer (i15, EDAN Shenzen, China) and coagulation analyzer (Sysmex CS-2500, Sysmex Europe, Norderstedt, Germany), respectively.

Additionally, the quantification of the biomolecule content of CB-PL and CB-PPP was performed using commercial ELISA kits. Specifically, the determination of PDGF-BB, TGF-β1, FGF, VEGF-A, HGF, indoleamine 2.3 dioxygenase (IDO), and prostaglandin E2 (PGE2) was performed following the manufacturer’s instructions (OriGene Technologies, Rockville, MD, USA). Plasma samples (*n* = 25), obtained from CBUs after single centrifugation at 780 g for 15 min were served as a control group for the biomolecule quantification assay.

### 2.5. Isolation of Corneal Epithelial Cells

To further evaluate the effect of CB-PL and CB-PPP, corneal epithelial cells (CECs) were isolated from Wistar rats and used for the performed experimental procedures.

All care and handling of the animals was provided according to the Guide for the Care and Use of Laboratory Animals of Biomedical Research Foundation Academy of Athens (BRFAA), conformed to the Directive 2010/63/EU of the European Parliament, and the ethical standards of the Greek National Ethical Committee and approved by our institution’s ethical board (Reference No. 1121, 10 June 2021).

The CECs were derived from male Wistar rats (*n* = 5), 3–4 months old, weighing 200 g. For CEC isolation, the animals were sacrificed, and the entire eye was removed using sterile instruments. Then, the disc of corneal tissue was dissected (including the limbus region). The disc was enzymatically treated using collagenase type I (Clostridium histolyticum) 1 mg/mL (Sigma-Aldrich, Darmstadt, Germany) and the samples were incubated at 37 °C for 4 h. Inactivation of collagenase was performed, followed by centrifugation at 500 g for 6 min. The supernatant was discarded, and the pellet was resuspended in a complete cell culture medium and placed into a 25 cm^2^ flask. The CECs expanded and passaged until the desired cell number was reached for the rest of the experiments. All CECs were at passage 1 when used for the rest of the experimental procedures.

The complete cell culture medium, used in this study, consisted of A-Minimum Essentials Medium (α-MEM, Gibco, Thermo Fisher Scientific, Waltham, Massachusetts, USA), supplemented with 15% *v/v* Fetal Bovine Serum (FBS, Gibco, Thermo Fisher Scientific, Waltham, MA, USA), 1% Penicillin-StreptomyciFn (Gibco, Thermo Fisher Scientific, MA, USA), and 1% L-glutamine (Gibco, Thermo Fisher Scientific, Waltham, MA, USA). The cell culture medium was changed twice a week in each sample, and kept at 4 °C, for no more than 14 days.

### 2.6. Effect of Cord Blood Platelet Lysate and Cord Blood Platelet Poor Plasma on Corneal Epithelial Cells Characteristics

In this study, the impact of CB-PL and CB-PPP on CECs characteristics was evaluated. Specifically, CECs morphology, viability, and cell proliferation were determined. To evaluate the cell morphology, the cells were initially cultured with the cell culture medium until 80% confluence was observed. Then, the medium was removed, the cell cultures were extensively washed with PBS 1× (Gibco, Thermo Fisher Scientific, Waltham, MA, USA), and finally, a culture medium containing either 20% *v/v* CB-PL or CB-PPP was added. CECs cultured with the complete culture medium (with 15% *v/v* FBS, 1% *v*/*v* P-S and 1% *v/v* L-glu) or cultured only with α-MEM (Gibco, Thermo Fisher Scientific, Waltham, Massachusetts), served as a positive and negative control group, respectively. The cell morphology in all groups was evaluated by measurements of length and width, and finally the cell elongation index (CEI) was determined. CEI was defined as the ratio of length to width.

Images were acquired using a Leica DM L2 light microscope (Leica Microsystems, Weltzar, Germany) and processed with ImageJ 1.46r (Wane Rasband National Institutes of Health, University of Wisconsin, Madison, WI, USA).

The evaluation of metabolic activity was performed using the ADP/ ATP ratio kit, following the manufacturer’s instructions (MAK189, Sigma-Aldrich, Darmstadt, Germany). The method relied on the measurement of the light intensity, which was specific to ATP concentration. The reaction performed is the following:

ATP + D-Luciferin + O_2_ → oxyluciferin + AMP + PP_i_ + CO_2_ + light (2)

Then, conversion of ADP to ATP is performed which further reacted with the D luciferin. The determination of this (second) light intensity corresponded to the total ADP and ATP concentration. The determination of the different light intensities was performed using the luminometer (Lucy 1 Anthos, Luminoskan, Labsystmems, Biocompare, South San Francisco, CA, USA). The corresponded light intensity was expressed as the number of relative light units (RLUs). The following formula was used for the final determination of the ADP/ATP ratio.

ADP/ATP ratio = (RLU C−RLU B) ÷ RLC A (3)

where RLU A is the initial luminescence measurement after the addition of the ATP reagent. RLU B is the luminescence measurement after 10 min of incubation, and RLU C is the measurement of light intensity after the addition of ADP reagent.

For the determination of the ADP/ ATP ratio, the CECs were seeded at a density of 1 × 10^5^ cells in 24 well plates with 1 mL of culture medium containing either 20% *v/v* CB-PL or 20% *v/v* CB-PPP and remained seeded for 96 h.

The proliferation of CECs was also estimated in the present study at specific time points (TP, 24, 48, 72, and 96 h). Each time, the CECs were detached using 0.025% Trypsin-EDTA (Gibco, Thermo Fisher Scientific, Waltham, MA, USA) solution for 10 min at 37 °C. In each TP, total cell number, cell doubling (CDT), cumulative population doubling (PD), and cell viability were determined.

The CDT was calculated based on the following equation:

CDT = log_10_(*N*/*N*_0_) ÷ log_10_(2) × T (4)

The PD was estimated based on the equation below:

PD = log_10_(*N*/*N*_0_) ÷ log_10_(2) (5)

where *N* was the number of cells at each passage, *N*_0_ was the number of initially plated CECs and T was the culture duration in hours.

CPD was obtained by adding all the PD values from each TP.

Total cell counting and viability estimations were performed using an automated system (Countess II FL Automated Cell Counter, Thermo Fisher Scientific, Waltham, MA, USA) with Trypan blue stain (Invitrogen, Thermo Fisher Scientific, Waltham, MA, USA).

The aforementioned experimental procedures involved 4 groups, (a) CECs (*n* = 5) expanded using α-ΜΕΜ supplemented with 20% *v/v* CB-PL (Group A), (b) CECs (*n* = 5) expanded using α-MEM supplemented with 20% *v/v* CB-PPP (Group B), (c) CECs (*n* = 5) expanded using α-MEM supplemented with 15% *v/v* FBS, 1% *v/v* P-S and 1% *v/v* L-glu (Sigma-Aldrich, Darmstadt, Germany, positive control group), and (d) CECs (*n* = 5) cultured only with α-MEM (negative control group).

### 2.7. Scratch Wound Assay

The migration capacity of CECs was evaluated, by performing the scratch wound assay. CECs were initially cultured in 24 well plates (Costar Corning Life, Canton, MA, USA) with the cell culture medium, upon confluence (80–90%) reached. Then, the culture medium was totally removed, followed by brief washes with PBS 1× (Gibco, Thermo Fisher Scientific, Waltham, MA, USA).

Finally, a gap occurred in the middle of the wells using 1 mL pipette tip, and the addition of 1 mL of α-MEM contained either 20% of CB-PL or CB-PPP, was performed. In positive and negative control groups, the same procedure as mentioned above was followed, and finally, either 1 mL of complete cell culture medium (positive control group) or 1 mL of α-MEM (negative control group) was added to the CECs cultures, respectively.

The migration of CECs was monitored until complete gap closure performance, and images were acquired at specific time points, using the inverted light microscope (Leica, DMII, Leica Microsystems, Wetzler, Germany) and processed with IC Capture v 2.4 software (Imaging Source, Bremen, Germany).

### 2.8. Characterization of Corneal Epithelial Cells Using Immunoassays

Culture-expanded CECs with culture medium containing either 20% *v/v* CB-PL or 20% *v/v* CB-PPP were further characterized using immunoassay experiments. Specifically, the determination of CECs proliferation was performed using immunoassays against phospho MAP kinase (Sigma-Aldrich, Darmstadt, Germany) and 5′ bromo 2′ dexyuridine (BrdU), Sigma-Aldrich Darmstadt, Germany). For the performance of the above assays, the CECs were placed on culture slides in several 5 × 10^5^ cells. The same procedure was repeated for the CECs of positive and negative control groups.

Specifically, immunohistochemistry against phospho MAP kinase was performed. For this purpose, the EnVision FLEX Mini kit, high pH (Agilent Technologies, Santa Clara, CA, USA) was used, following the manufacturer’s instructions. Initially, the culture slides containing the CECs were fixed with 10% *v/v* neutral formalin buffer, peroxidase blocked, followed by the antigen retrieval. Then, the addition of anti-phospho MAP kinase (1:1000) was performed, followed by the addition of mouse secondary IgG antibody (1:500). Then, the slides remained at 4 °C for more than 8 h (overnight). Then, DAB was added to all groups, and the samples remained in a dark place until color development. Finally, Harry’s hematoxylin staining (Sigma-Aldrich, Darmstadt, Germany) was performed.

Images were acquired using Leica DM LS2 microscope (Leica microsystems, Wetzlar, Germany) and processed with the IC Capture v2.4 software (Imaging Source, Bremen, Germany).

Indirect immunofluorescence experiments were performed to validate the expression of both phospho MAP kinase and Brd, in CEC of all study groups. The culture slides contained the CECs were initially fixated using the 10% *v/v* neutral formalin buffer (Sigma-Aldrich, Darmstadt, Germany) for 10 min. Then, we performed antigen retrieval using TRIS- Ethylenediaminetetraacetic acid (EDTA) buffer pH 9 (10 mM Tris Base, 1 mM EDTA, 0.05% Tween 20, Sigma-Aldrich, Darmstadt, Germany), and blocking with 5% Bovine Serum Albumin (BSA, Sigma-Aldrich, Darmstadt, Germany) was performed. Firstly, the addition of monoclonal antibody against phospho-MAP kinase (1:1000, incubated at 37 °C for 2 h), and secondly addition of monoclonal antibody against BrdU (1:000, incubated at 37° for additional 2 h) was performed, following by the addition of anti-rabbit and anti-mouse IgG (1:50, incubated for maximum 1 h each) antibodies, respectively. The slides were incubated for 1 h with the secondary monoclonal antibodies and glycerol mounted. Observation of the results was performed using the fluorescent microscope. Images were acquired with Leica SP5 II microscope equipped with LAS Suite v2 software (Leica, Microsystems, Wetzlar, Germany).

### 2.9. Flow Cytometric Analysis of Corneal Epithelial Cells

To further determine the impact of either the CB-PL or CB-PPP on CEC properties, flow cytometric analysis was performed. Common CD markers for CECs, including CD29, CD73, CD340, and CD44 were determined. All monoclonal antibodies used for the flow cytometric analysis of the CECs were purchased from the Beckton Dickinson (BD Biosciences, NJ, USA).

The CD markers were labelled with fluorescein (CD29-FITC), peridinin-chlorophyll-protein (CD73-PerCP) and phycoerythrin (CD340-PE and CD44-PE). Briefly, the CECs of passage 3 (P3) were detached, counted, and placed in 5 mL tubes at a density of 5 × 10^4^ cells/mL. Then, 20 μL of each CD marker was added, followed by a brief vortex and incubation at room temperature (RT) for 30 min in dark. Then, centrifugation of the samples was performed at 500 g for 6 min, followed by removal of the supernatant and reconstitution with 1 mL of PBS 1x (Gibco, Thermo Fisher Scientific, Waltham, MA, USA) was performed. Finally, the samples were loaded to the flow cytometry (FACS Calibur, BD Biosciences, Franklin Lakes, NJ, USA).

All samples were initially analyzed through front (FSC) and side (SSC) scatter, and then adjustment of compensation settings to each CD marker was performed. On average several 10,000 all events were acquired for each sample. In this assay, 4 study groups were included: (a) CECs expanded with culture medium containing 20% *v/v* CB-PL, (b) CECs expanded with culture medium containing 20% *v/v* CB-PPP, (c) CECs expanded with the complete culture medium (15% *v/v* FBS) served as the positive control group and (d) CECs cultured with α-ΜΕΜ (Sigma-Aldrich, Darmstadt, Germany) only served as the negative control group.

### 2.10. Gene Expression Analysis of Corneal Epithelial Cells

Gene expression analysis of CECs was also performed. In this manner, CECs of all study groups were evaluated for the expression of *Collagen chain A 1* (*Col1A1*), *Syndecan type 1*, *Perlecan*, and *b-actin* (housekeeping gene). The total RNA was initially isolated using the TRI reagent (Sigma-Aldrich, Darmstadt, Germany), according to the manufacturer’s instructions. The isolated mRNA was quantified, and 800 ng were transcribed using the reverse transcription (RT)- polymerase chain reaction (PCR, Qiagen, Hilden, Germany). Then, the complementary DNA (cDNA) was used for the amplification with the primers listed in Table 1. The PCR involved the following steps: (1) initial denaturation at 95 °C for 15 s, (2) denaturation at 94 °C for 30 s, (3) annealing at 60–61 °C for 90 s and (4) extension at 72 °C for 3 min. The same procedure was repeated for another 34 cycles. Finally, the PCR products were analyzed with agarose gel (1% *v/v*, Sigma-Aldrich, Darmstadt, Germany) electrophoresis (Bio-Rad Laboratories, Hercules, CA, USA).

### 2.11. Functional Protein Analysis

To further understand the molecular interactions between the biomolecules released (either by CB-PL or CB-PPP) and specific cell functions (migration, proliferation, and tissue remodeling), functional protein analysis of the involved signaling pathways was performed. For this purpose, the understanding of the biomolecule interactions with cell functions was performed using the online tool STRING—functional protein network association (https://string-db.org, accessed on 15 June 2022). The bioinformatic analysis was applied using the initially determined biomolecules of CB-derivatives including the TGF-β1, FGF, PDGF, VEGFA, HGF, and IDO. Verification of our initial obtained network pathways was performed by KEGG Pathway Database (https://www.genome.jp/kegg/pathway.html (accessed on 17 June 2022) and Cytoscape v3.9.1 (Cytoscape, Institute of Systems Biology in Seattle, Seattle, WA, USA), guiding us to propose a potential model.

### 2.12. Statistical Analysis

GraphPad Primsm v6 (GraphPad Software, San Diego, CA, USA) was used for the performance of the statiscal analysis in this study. A nonparametric Kruskall–Wallis test was used to find the statistically significant differences between the study groups. To further validate our results we also applied the unpaired nonparametric Mann-Whitney U test. Statistically significant differences were considered when *p*-value was less than 0.05. Indicated values were represented as mean ± standard deviation.

## 3. Results

### 3.1. Characterization of Cord Blood Derivatives

The initial volume of the CBUs used in the present study was 139.4 ± 5.8 mL. The concentration of WBCs, RBCs, and HgB was 9.2 ± 1.9 × 10^3^/μL, 3.6 ± 1.1 × 10^3^/ μL, and 11.3 ± 1.1 g/dL, respectively. The concentration of PLTs in unprocessed CBUs was 202.2 ± 27.9 × 10^3^ / μL. This data confirmed that the CBUs did not fulfill the minimum criteria for processing, cryopreservation and release for administration, outlined by the -FACT-NetCord (Table S1), which can be further processed for the production of CB- derivatives. In this manner, the CB-PC and CB-PPP production was followed. The concentration and total number of PLTs in CB-PC were 1120.3 ± 70.4 × 10^3^/μL and 12.1 ± 1.5 × 10^9^, respectively. The recovery rate of PLTs in PC was 42.3 ± 8.2%. The mean volume of the produced CB-PC was 10.4 ± 1.1 mL. Furthermore, CB-PC was characterized by low rates of WBCs, RBCs, and HgB. Detailed characteristics of CB-PC samples can be observed in Table 2.

With respect to CB-PPP samples, the concentration of WBCs, RBCs, HgB, and PLTs was extremely low compared to the CB-PC samples. The net volume of the CB-PPP was 20.7 ± 2.6 mL and the PLT number was 0.3 ± 0.1 × 10^9^. More details on the CB-PPP quality characteristics can be observed in Table 1. Statistically significant differences regarding the concentration of WBCs, RBCs, PLTs, volume, and recovery rates were observed between CB-PC and CB-PPP (*p* < 0.001).

The above data indicated the successful production of both CB-PC and CB-PPP from the whole CBUs. Furthermore, the CB-PC samples were stored at −80 °C and typically after some days (at least after 1 day), were rapidly thawed and used for the evaluation.

Both CB-PL and CB-PPP were passed through a sterilization filter (0.22 μm) before use, eliminating the WBCs (0 × 10^3^/μL), RBCs (0 × 10^6^/μL), HgB (0 g/dL), and PLTs (0 × 10^3^/μL). Finally, only the growth factors derived from platelets’ lysis remained in the CB-PL. All CBUs, CB-PL, and CB-PPP products of this study were discovered to be negative for active viral and microorganism infections (Table S2).

### 3.2. Quality Characteristics of Cord Blood Platelet Lysate and Cord Blood Platelet Poor Plasma

To evaluate the production of high-quality CB-PL and CB-PPP, the following characteristics were defined. Both CB- derivatives were tested for pH, lactate, glucose, PCO_2_, PO_2_, prothrombin time test (PT), activated partial thromboplastin time (APTT), and fibrinogen. Description of all tested parameters for CBUs, CB-PL, and CB-PPP are located in Table 3.

Finally, the CBUs did not demonstrate any great variances for the above parameters compared to CB-PL and CB-PPP, and therefore no statistically significant differences were observed between all groups.

### 3.3. Biomolecule Quantification

Further evaluation of the CB-PL and CB-PPP involved the quantification of the secreted biomolecules. For this purpose, a specific set of growth factors, including the TGF-β1, PDGF-BB, FGF, VEGF-A, and HGF, were quantified in this study.

**Figure 1:**
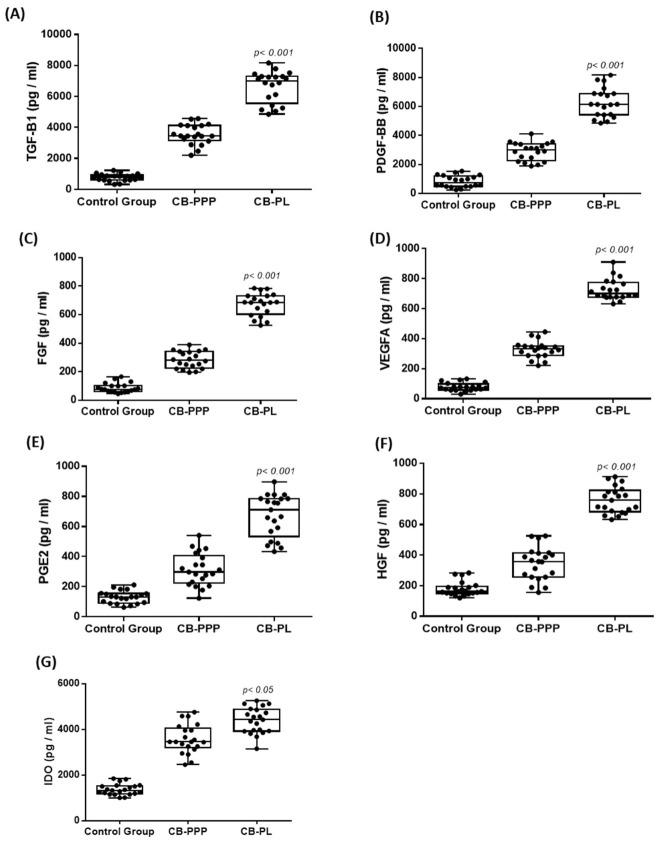
Quantification of biomolecule secretion in CB-PL and CB-PPP. The evaluation of biomolecule secretion involved the quantification of TGF-β1 (**A**), PDGF-BB (**B**), FGF (**C**), VEGF-A (**D**), PGE2 (**E**), HGF (**F**), and IDO (**G**). A Kruskal–Wallis test indicated statistically significant differences between the control group, CB-PL, and CB-PPP with respect to the TGF-β1 (*p* < 0.001), PDGF-BB (*p* < 0.001), FGF (*p* < 0.001), VEGF-A (*p* < 0.001), HGF (*p* < 0.001), PGE2 (*p* < 0.001), and IDO (*p* < 0.05), were observed.

The determined levels of TGF-β1, PDGF-BB, FGF, VEGF-A, and HGF for the CB-PL were 6628 ± 985 pg/mL, 6268 ± 980 pg/mL, 672 ± 77 pg/mL, 726 ± 68 pg/mL, and 760 ± 84 pg/mL, respectively. The levels of the growth factors for the CB-PPP were 3593 ± 629 pg/mL, 2907 ± 605 pg/mL, 282 ± 58 pg/mL, 327 ± 56 pg/mL, and 343 ± 106 pg/mL, respectively (Figure 1). Statistically significant differences were observed between the CB-PC and CB-PPP, with respect to the levels of TGF-β1 (*p* < 0.001), PDGF-BB (*p* < 0.001), FGF (*p* < 0.001), VEGF-A (*p* < 0.001), and HGF (*p* < 0.001).

Aside from these growth factors, other molecules with known immunoregulatory properties were also evaluated in CB-PL and CB-PPP. Specifically, the levels of PGE2 and IDO were 673 ± 137 pg/mL, 314 ± 105 pg/mL, 4383 ± 553 pg/mL, 3568 ± 621 pg/mL for CB-PL, and CB-PPP, respectively (Figure 1). Statistically significant differences between PGE2 and IDO were observed in CB-PL and CB-PPP (*p* < 0.05).

In this study, plasma samples were also evaluated for the secretion of the above biomolecules and served as the control group. However, the levels of TGF-β1, PDGF-BB, FGF, VEGF-A, HGF, PGE2, and IDO were extremely low compared to CB-PC and CB-PPP (Figure 1). Statistically significant differences between the control group, CB-PC and CB-PPP regarding the levels of TGF-β1 (*p* < 0.001), PDGF-BB (*p* < 0.001), FGF (*p* < 0.001), VEGF-A (*p* < 0.001), HGF (*p* < 0.001), PGE2 (*p* < 0.001), and IDO (*p* < 0.05), were observed.

### 3.4. Isolation and Characterization of the Corneal Epithelial Cells

**Figure 2:**
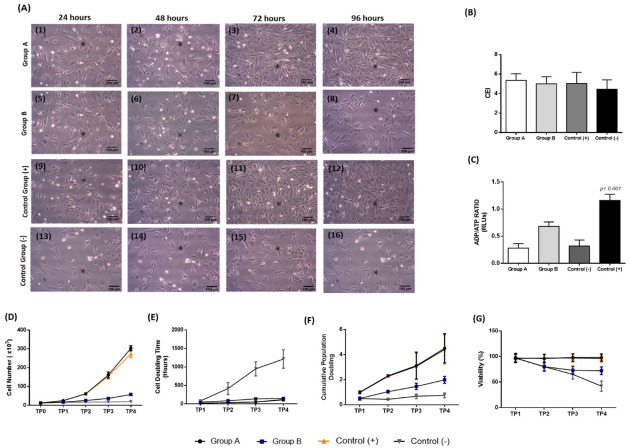
Evaluation of CEC characteristics utilizing different experimental approaches. (**A**) Morphological features of the CECs of group A (A1–A4), (**B**) (A5–A8), positive control group (A9–A12), and negative control group (A13–A16). CECs of group A and positive control group were culture expanded and proliferated well until confluence was observed after 96 h. CECs of group B and negative control group did not proliferate with the same rate compared to the above groups and eventually no confluence was observed after 96 h. All images acquired with original magnification 10x and scale bars 100 μm. Evaluation of the CEI (**B**) and ADP/ATP ratio (**C**) of CECs. Evaluation of quality characteristics of CECs, including cell number (**D**), CDT (**E**), CPD (**F**), and viability (**G**). CECs of group A and positive control group characterized by better expansion and proliferation properties compared to those of group B and negative control group. Statistically significant differences were observed in ADP/ATP ratio (*p* < 0.001), cell number (*p* < 0.001), CDT (*p* < 0.05), cumulative PD (*p* < 0.05) and viability (*p* < 0.001), between all groups. Group A: CECs cultured with α-ΜΕΜ supplemented with 20% *v/v* CB-PL), Group B: CECs cultured with α-ΜΕΜ supplemented with 20% *v/v* CB-PPP, Control group (+): Positive control group, CECs cultured with complete culture medium, Control group (−), negative control group: CECs cultured only with α-MEM.

CECs were successfully isolated from the corneal tissue and expanded under in vitro results. Specifically, CECs of group A presented similar morphological features as the cells in the control group (cultured with the complete cell culture medium, Figure 2A). Conversely, CECs of group B were expanded with a slower rate compared to group A and positive control group (Figure 2A). However, CECs in all study groups exhibited a similar cell elongation index (CEI, Figure 2B).

The metabolic activity of CECs was determined using the ADP/ATP ratio. CECs of group A and positive control group exhibited 0.28 ± 0.07 and 0.32 ± 0.09 RLUs (Figure 2C). CECs of group B and the negative control group were 0.68 ± 0.07 and 1.16 ± 0.101 RLUs, respectively. CECs of group B exhibited a higher ADP/ATP ratio compared to group A and positive control group (Figure 2C). However, the RLUs of all study groups were statistically lower compared to negative control group (*p* < 0.001). Statistically significant differences in RLU content were observed between all study groups (*p* < 0.001).

To comprehensively evaluate the effect of both CB-PL and CB-PPP in CECs features, the determination of cell number, CDT, CPD, and viability was also performed. Specifically, CECs from group A and the positive control group successfully expanded after 96 h and reached a total number of 3 ± 0.1 × 10^6^ and 2.7 ± 0.1 × 10^6^ cells, respectively, while CECs of group B and the negative control group were 0.5 ± 0.1 × 10^6^ and 0.2 ± 0.1 × 10^6^ cells, respectively (Figure 2D). Similarly, the CDT and CPD of CECs after 96 h of the groups A and B were 110 ± 27 h, 4.5 ± 1.1 and150 ± 18 h, 1.9 ± 0.2, respectively. The CDT and CPD of CECs from the positive and negative control groups were and 123 ± 29 h, 4.4 ± 1.7 and 1214 ± 250 h, 0.7 ± 0.1, respectively (Figure 2E and F). The viability of CECs of groups A and B, in addition to positive and negative control groups after 96 h was 98 ± 7%, 72 ± 8%, 96 ± 7% and 42 ± 10%, respectively (Figure 2E). Statistically significant differences regarding the cell number (*p* < 0.001), CDT (*p* < 0.05), CPD (*p* < 0.05) and viability (*p* < 0.001) were observed between all groups.

### 3.5. Scratch Wound Assay

**Figure 3:**
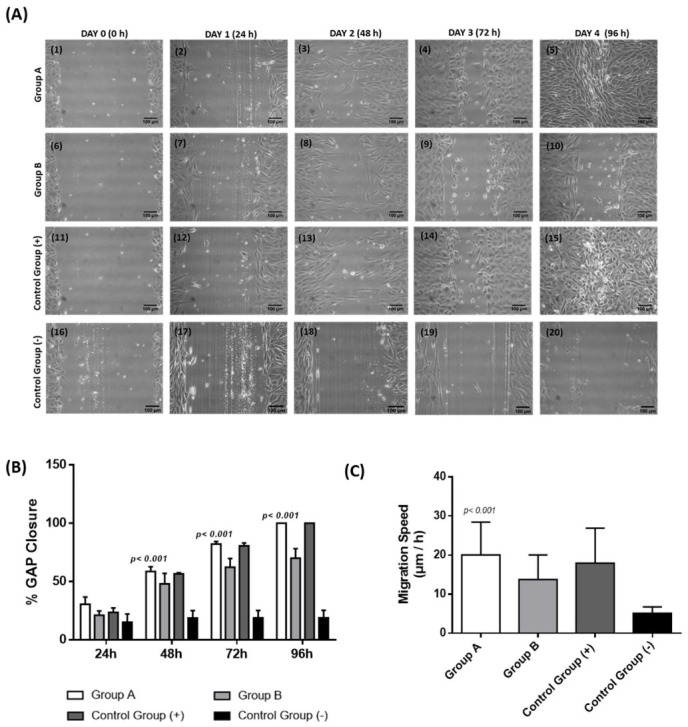
Performance of scratch-wound assay for the determination of the migratory capacity of CECs (**A**). Specifically the migratory capacity of CECs from group A (A1–A5), group B (A6–A10), positive control group (A11–A15), and negative control group (A16–A20), was monitored. All images were acquired with original magnification 10× and scale bars 100 μm. Total gap closure was observed in CECs of group A and positive control group after 4 days of the initial cultivation. Conversely, CECs of group B migrated but did not achieve the gap closure after 4 days of the initial cultivation. Also, CECs of negative control group presented slow proliferation potential and did not fill the gap. Diagrams portray the percentage of gap closure (**B**) and migration speed of CECs (**C**). Specifically, CECs of group A and positive control group indicated good migratory capacity and the gap was successfully closed after 96 h, whereas CECs of group B and negative control group did not fill the gap after 96 h. A Kruskal–Wallis test indicated statistically significant differences regarding the gap closure (*p* < 0.001) and migration speed (*p* < 0.001) between all groups. Group A: CECs cultured with α-ΜΕΜ supplemented with 20% *v/v* CB-PL), Group B: CECs cultured with α-ΜΕΜ supplemented with 20% *v/v* CB-PPP, Control group (+): Positive control group, CECs cultured with complete culture medium, Control group (−), negative control group: CECs cultured only with α-MEM.

The evaluation of the regenerative potential and migration capacity of CECs cultured either with the CB-PL or CB-PPP was performed using the scratch-wound assay (Figure 3). The greatest migration capacity was presented by the CECs of group A. Total gap closure was observed in group A after 4 days from the initial gap occurrence. Also, CECs of the positive control group demonstrated good migratory capacity, and the gap was completely closed after 4 days. However, CECs of group A were characterized by better morphological features compared to the positive control group. Conversely, CECs of group B exhibited low migration ability and after 96 h the initial gap did not close.

Complete gap closure was observed only in group A and the positive control group after 96 h, whereas group B and the negative control group did not achieve full gap closure (Figure 3B).

Also, the migration speed of the proliferated CECs was estimated in all study groups. Briefly, the highest migration speed was presented in group A (20 ± 8 μm/h) and positive control group (18 ± 9 μm/h). Conversely, the migration speed of group B and negative control were 14 ± 6 μm/h and 5 ± 2 μm/h, respectively (Figure 3C). An ANOVA test indicated significant differences in the gap closure (*p* < 0.001) and migration speed (*p* < 0.001) between all study groups.

### 3.6. Immunostaining, Gene Expression Analysis and Immunophenotype of Corneal Epithelial Cells

**Figure 4:**
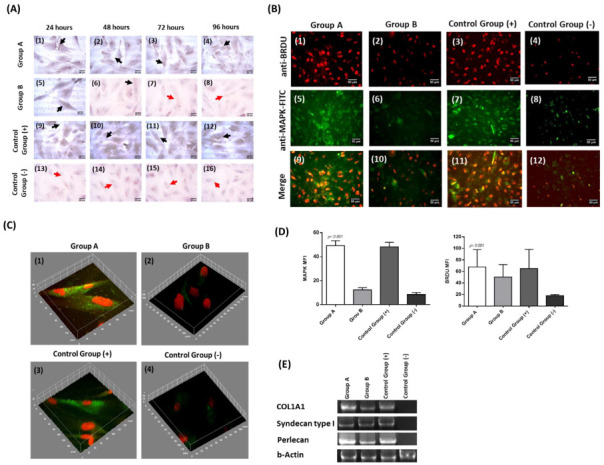
Immunostaining and gene expression analysis of CECs. Immunohistochemistry against MAP kinase in CECs of all groups (**A**). Specifically, differences in MAP kinase expression levels were detected between groups A (A1–A4), group B (A5–A8), positive control group (A9–A12) and negative control group (A13–A16). Black and red arrows in images A1–A16 indicated the presence and absence of MAP kinase, respectively. All images were acquired with original magnification 40x and scale bars 25 μm. Performance of indirect immunofluorescence against MAP kinase in combination with BrdU in CECs (**B**). Indirect immunofluorescence against MAP kinase and BrdU in CECs of group A (B1, B5, B9), group B (B2, B6, B10), positive control group (B3, B7, B11), and negative control group (B4, B8, B12). All images were acquired with original magnification 20× and scale bars 50 μm. 3D reconstruction models of CECs obtained from the indirect immunofluorescence against MAP kinase and BrdU (**C**). 3D reconstruction images of CECs from group A (C1), group B (C2), positive control group (C3), and negative control group (C4). Mean fluorescence intensity of MAP kinase and BrdU of CECs from all study groups (**D**). Molecular analysis and detection of *Col1A1*, *Syndecan type 1*, *Perlecan* and *b-actin* of CECs from all study groups (**E**). Group A: CECs cultured with α-ΜΕΜ supplemented with 20% *v/v* CB-PL), Group B: CECs cultured with α-ΜΕΜ supplemented with 20% *v/v* CB-PPP, control group (+): positive control group, CECs cultured with complete culture medium, control group (−), negative control group: CECs cultured only with α-MEM.

To validate further the proliferation capacity of CECs, specific immunoassays using the phospho-MAP kinase and BrdU were performed. Immunohistochemistry results indicated the expression of MAP kinase in group A and the positive control group (Figure 4A). However, group B and the negative control group were characterized by lower expression of MAP kinase (Figure 4A). After 96 h of cultivation, CECs of group A and positive control group were stable expressed the MAP kinase. Conversely, the MAP kinase expression was barely detected in CECs of group B and negative control group. (Figure 4A).

Moreover, immunofluorescence against MAP kinase in combination with BrdU was also performed, to further validate the proliferation of the CECs (Figure 4B). Specifically, BrdU and MAP kinase were detected in all study groups. However, group A and the positive control group were characterized by higher signal intensity for both markers compared to group B and the negative control group. Furthermore, co-localization of the BrdU and MAP kinase was successfully detected in group A and in positive control group (Figure 4C,D).

Aside from the immunostaining results, gene expression analysis indicated that CECs from groups A, B, and positive control successfully expressed the *Col1A1*, *Syndecan Type I*, and *Perlecan* (Figure 4E). CECs of the negative control group did not express the above genes.

**Figure 5:**
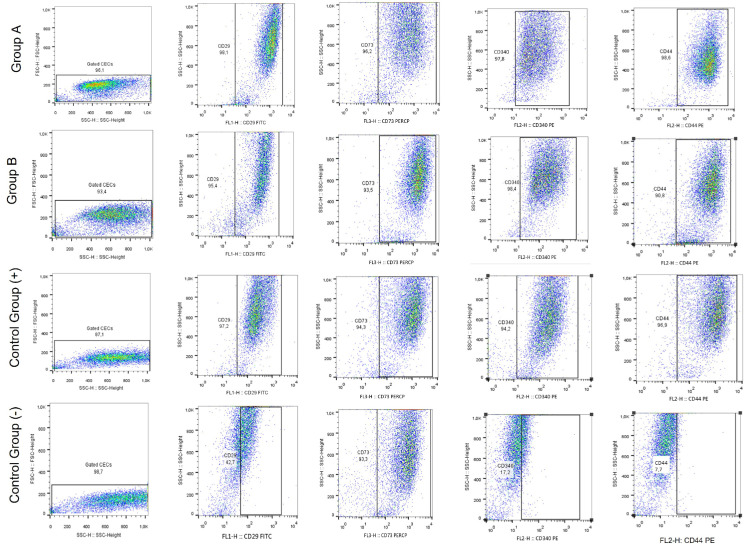
Flow cytometric analysis of CECs. CECs of all groups were examined for the expression of CD29, CD73, CD340, and CD44. Initially, to determine the cellularity and granulation, CECs were evaluated for the parameters of front scatter (FSC) and side scatter (SSC). Then, the CECs were analyzed further for the expression of CD29, CD73, CD340, and CD44. Group A: CECs cultured with α-ΜΕΜ supplemented with 20% *v/v* CB-PL), Group B: CECs cultured with α-ΜΕΜ supplemented with 20% *v/v* CB-PPP, control group (+): positive control group, CECs cultured with complete culture medium, control group (−), negative control group: CECs cultured only with α-MEM.

To further validate the effect of CB-PL and CB-PPP on CECs properties, flow cytometric analysis for CD29, CD73, CD340, and CD44 was performed. These CD markers are strongly associated with the activation, adhesion, and migration properties of CECs. Importantly, immunophentoypic analysis indicated that CECs of groups A, B, and positive control for CD29, CD73, CD340, and CD44 expressed >95% (Figure 5). Conversely, CECs of negative control group exhibited different expression patterns for the same markers. Indeed, the levels of CD29, CD340, and CD44 were 40 ± 3%, 92 ± 3%, 17.1 ± 2% and 7.2 ± 3%, respectively (Figure S1). Statistically significant differences regarding the percentage of CD markers expression for CD29 (*p* < 0.01), CD340 (*p* < 0.01) and CD44 (*p* < 0.01) between the negative control group and the rest of the groups.

### 3.7. Functional Protein Analysis

**Figure 6:**
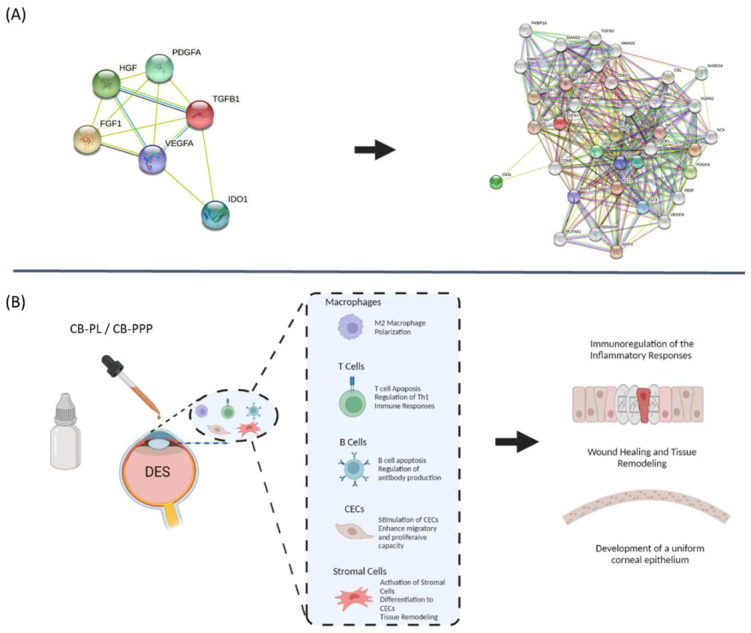
Tissue remodeling model of damaged corneal surface due to DES. (**A**) Functional protein analysis of CB-PL and CB-PPP using the online tool STRING (https://string-db.org, 15 June 2022). (**B**) Proposed model relied on the functional analysis data. DES is characterized an inflammatory process which can further lead to corneal surface damage. After the application of CB-PL/CB-PPP, the secreted proteins can ameliorate the activated immune responses through macrophage polarization, T and B cell immunomodulation, and apoptosis. Then, CECs of the wounded region can be re-activated due to growth factor presence, resulting in advanced adhesion, proliferation, and production of tissue remodeling proteins.

Functional protein analysis was performed to further evaluate the interactions between the biomolecules derived from the CB-PL and CB-PPP with specific cellular processes. Functional analysis revealed a great number of different interactions, where more than 30 proteins were identified and associated. Among them, significant signaling pathways that lead to specific intracellular functions, such as promoting the cell adhesion, migration, and differentiation, were revealed. Among them, the TGF-β1 signaling pathway seems to share many close interactions with other proteins, such as CAVEOLIN-1 (CAV1), ENDOGLIN (ENG), CADHERIN-1 (CDH1), and CADHERIN-5 (CDH5). Moreover, strong associations have been discovered between SEMAPHORIN 3A (SEMA3A), NEUROPILIN-1 (NRP1), PLEXIN-A1 (PLXNA1), and VEGFA, VEGFB, FGF, PDGFA. Also, IDO and HGF appear to exhibit important interactions with the TGF-β1 signaling pathway (Figure 6).

The identified proteins were closely related with cell migration, adhesion, proliferation, and tissue remodeling, leading to beneficial wound healing properties.

However, other interactions revealed also the potential immunomodulatory action of both CB-PL and CB-PPP, which can be exerted through the activation of SEMAPHORIN 3A, PLEXIN A1, HGF, and IDO (Table S3).

## 4. Discussion

The application of specific blood derivatives such as the PL and PPP in wound healing applications has attracted the attention of the scientific community and could be considered as potential substitutes for the gold standard method (artificial tear drops, lubricants, and immunosuppressor eye drops) daily, worldwide [35]. PL and PPP, due to their regenerative potential mediated by the exerted biomolecule content, can share key therapeutic effects in different fields of medicine [36]. Considering that PL and PPP can enhance the wound healing and tissue regeneration processes, these products may exhibit beneficial properties in ophthalmic applications [37]. Ophthalmic disorders such as SS and cGVHD may result in DES development, which can lead to increased damage to the corneal surface and the resident epithelial cells [38,39]. The above events eventually lead to cornea malfunction and total vision loss [38,39]. Therefore, different approaches, including the topical applications of artificial tears, lubricants, etc. are mostly preferred to reduce the damage [6,7,8,9,16]. However, these commercial products are limited to regenerative potential, due to the absence of wound healing mediators such as growth factors and tissue regenerative proteins [6,7,8,9,16,40]. Conversely, accumulating evidence from the literature indicated that CB derivatives are characterized by a great content of biomolecules [18,19]. For this reason, the CB products have been used with success in patients suffering from skin lesions and extended wounds [23,24,25,26,27,28].

Accounting for these considerations, the topical application of eye drops produced either from CB-PL or CB-PPP may reverse the damage, thus causing a positive therapeutic outcome in patients suffering from DES.

In this study, initially, we evaluated the quality characteristics of the CB-PL and CB-PPP. No significant differences regarding the pH, lactate, glucose, PCO_2_, PO_2_, PT, APTT, and fibrinogen content were observed between these two CBderivatives. Then, evaluation of the growth factor content of CB-PL and CB-PPP was performed. Both products contained a specific number of biomolecules, including growth factors such as the TGF-β1, PDGF-BB, FGF, VEGFA, and HGF, and anti-inflammatory mediators such as PGE2 and IDO. However, CB-PL contained a significantly higher amount of the above proteins compared to the CB-PPP (*p* < 0.001). The above results regarding the growth factor content seem to be aligned with those that have been described in the literature [18,19,24,41]. In the study of Valentini et al. [24], comparable results regarding the levels of PDGF, VEGF, and FGF were presented. Also, in the study of Parazzi et al. [41] similar results to our study, with respect to the growth factor content in CB-PL and CB-PPP, were described. The results of the above studies seem to be comparable with those presented here, suggesting that both CB-derivatives contain a significant amount of growth factors. Furthermore, CB-PL and CB-PPP are contained the anti-inflammatory agents HGF, IDO, and PGE2 at specific concentrations. These identified proteins can exert specific therapeutic properties through their implication to various cell functions such as adhesion, migration, and proliferation, and can also serve as novel immunoregulatory agents [20,21,22,42,43,44]. It is widely recognized that HGF after binding to its receptor (Met) can promote downstream the activation of several signaling transducing proteins, such as Grb2, Stat3, and Gab1 [42,45]. These series of events leads to specific cellular functions, such as the promotion of angiogenesis, proliferation, survival, etc. [42,45]. Additionally, HGF may share also immunoregulatory properties in combination with other factors [42,45,46]. HGF mediates to the development of tolerogenic dendritic cells (tDCs) through autocrine IL-10 release [47]. IL-10 represents a pleiotropic cytokine, and its elevated levels are correlated with the suppression of the over-activated DCs, which can further promote the production of the tDCs [48]. The latter events are further related with the Th2 phenotype adaptation, which can lead to complete immunoregulation [49]. IDO is also characterized by key-specific immunoregulatory properties [43]. It has been indicated that IDO could induce the apoptosis of the over-activated T and B cells, through implication to kynurenine metabolism [50]. A growing field of evidence has indicated that IDO can induce the selective apoptosis of Th1 and not Th2 cells [43,49,50,51]. Considering the above facts, CB-PL and CB-PPP may exert a beneficial action to the wound healing process. Initially, the secreted growth factors can efficiently stimulate different cellular populations (progenitor and stem cells) to the injury site [20,21,22,42,43,44,45]. Moreover, the immunoregulatory biomolecules IDO, HGF, and PGE2 can contribute to immune-related processes, such as the M2 macrophage polarization and Th2 activation, which can further lead to advanced tissue remodeling of the injury site [42,43,44,45,46,47,48,49,50,51,52]. Therefore, these anti-inflammatory proteins can regulate the immune responses, providing enough time for the cell migration, proliferation, and differentiation at the wounded region [42,43,44,45,46,47,48,49,50,51,52].

The next step of this study involved the cultivation of CECs in presence in culture medium containing either 20% *v/v* CB-PL or 20% *v/v* CB-PPP, to further validate their beneficial properties on cells’ characteristics. CECs cultured with CB-PL successfully expanded after 96 h, whereas those cultivated with CB-PPP presented lower expansion rates (compared to the CB-PL expanded CECs). The use of PL as animal serum substitute has been also proposed previously, demonstrating promising results [53,54]. Indeed, stem cells such as the mesenchymal stromal cells (MSCs) expanded with PL obtained from peripheral blood, were clinically applied and characterized by high proliferation and viability rates [53,54,55] Atashi et al. [56] have provided sufficient evidence for the successful expansion of adipose-derived mesenchymal stem cells using autologous platelet-rich plasma. Conversely, there is limited evidence provided in the literature with respect to the potential application of CB-PL in CEC expansion and characteristics evaluation [57]. The results of this study indicated that CB-PL can promote the proliferation of CECs, comparably to the aforementioned studies. Moreover, the results of our study seem to align with other studies in the field. Specifically, Mishan et al. [58] and Seidelmann et al. [59] demonstrated that PL obtained from human peripheral blood can be used for the isolation and expansion of corneal stromal keratocytes and fibroblasts. Conversely, CB-PPP can support the CECs culture, however, limited cell expansion was observed. This may be explained by the lower biomolecule content of CB-PPP compared to CB-PL. These data further confirmed by experimental procedures indicated that CECs cultured with CB-PL presented higher cell number and viability and lower CDT and cumulative PD, compared to those cultured with CB-PPP.

To further evaluate the impact of CB-PL and CB-PPP in CECs expansion, a scratch-wound assay was performed. In a similar manner to previously published articles, CECs cultured with the medium contained 20% *v/v* CB-PL, were successfully expanded and the gap was completely closed after 4 days [18,60]. Conversely, CECs cultured with medium contained 20% *v/v* CB-PPP and proliferated but did not achieve complete closure of the initially occurred gap. To further assess the differences in CEC proliferation and migration, experimental assays involving the detection of MAP kinase expression between the different study groups were performed. Both immunohistochemistry and indirect immunofluorescence assays indicated high expression of MAP kinase in CECs cultured either with the CB-PL or the regular culture medium (positive control group). Conversely, CECs cultured with the CB-PPP expressed the MAP kinase at 24 h, however, after 48 h the expression levels of MAP kinase were significantly lower. CECs of the negative control group did not express the MAP kinase either in immunohistochemistry or in indirect immunofluorescence experiments.

It is known that a MAP kinase can be activated by several growth factors, such as TGF-β1 and VEGF, leading to the activation of the downstream signaling pathway (RAS-RAF-MEK-ERK), which eventually can promote specific cellular functions such as cell adhesion, migration, and proliferation [61,62]. Based on the results of our study, CB-PL is a rich source of several signaling-transducing growth factors, explaining the high proliferative and migratory capacity of cells. CB-PPP is characterized by less growth factor content compared to CB-PL. This may explain the fact that CECs expanded with the CB-PPP initially expressed in the MAP kinase and proliferated for a limited period of time. These results were further supported by evidence obtained from the negative control group. The MAP kinase, due to the absence of these factors, was not activated and could not be detected in the performed assays. To confirm the high proliferation rate of the CECs, indirect immunofluorescence against MAP kinase and BrdU was also performed. High expression of BrdU was evident in CECs cultured either with the CB-PL or the regular culture medium (positive control group). Moreover, using the 3D reconstruction models, colocalization of MAP kinase and BrdU was detected. Indeed, BrdU is incorporated rapidly in dividing cells during the S-phase of cell cycle, and is closely related with the cellular proliferation and expansion [63]. The results of this study clearly demonstrated that primarily CB-PL and CB-PPP can support the in vitro CEC cultures, thus it could be applied for the wound healing purposes in ophthalmology.

Additional evidence of the important role of MAP kinase in cell migration, proliferation and its close relationship with the TGF-β1 arose from the study of Terai et al. [64]. In this study, conditionally ablated transgenic mice for TGF-β receptor type II were generated to assess further the wound healing process of the damaged cornea epithelium [64]. The results of this study, indicated that mice lacking the TGF-β receptor, exhibited delayed and limited activation of MAP kinase. Moreover, in the study of Saika et al. [65], total blockage of the MAP kinase was applied, using the inhibitor SB202190. The common feature in both studies was the suppression of actin stress fibers at the migrating edge, resulting in the subsequent halt of CECs proliferation and migration to the occurred wound region [64,65]. These results clearly demonstrated the close function-relationship between the TGF-β and MAP kinase signaling pathway, suggesting that the high growth factor content of the CB-PL may favor the outlined cell functions. This also explains the greater proliferation and migratory potential of the CB-PL cultured CECs compared to those cultured with the CB-PPP.

Further confirmation of the beneficial effects of CB-PL and CB-PPP in CECs features was presented by the determination of specific gene expression. CECs of all study groups (except those of negative control group) successfully expressed important genes such as *Col1A1*, *Syndecan type I*, and *Perlecan*. In the literature, it has been demonstrated that CECs lacking the expression of syndecan type I and perlecan have been related to delayed wound repair, migration, and increased apoptosis [66]. Additionally, collagen represents a key extracellular protein, providing the appropriate mechanical resistance and function to the restored wound region [67]. The above results were further confirmed by the immunophenotype analysis of CECs using the flow cytometry. Only CECs of the negative control group presented low levels of expression for the CD29, CD340, and CD44. These proteins share great association with activation, adhesion, and migration. Conversely, CECs cultured with CB-PL, CB-PPP, and regular culture medium successfully expressed >90% the typical markers CD29, CD73, CD340, and CD44 [68]. Specifically, CD73 and CD340 are mostly related with CECs mobilization through activation of ΝF-kβ signaling pathway [68]. NF-kβ is an important promoter of the MAP kinase signaling pathway, indicating another positive implicating signaling pathway for CEC function. This could further support the evidence that the application of CB-derivatives is mostly related with beneficial outcome for CECs functions, such as migration, proliferation, and wound healing.

In the context of applying CB-derivatives for corneal wound healing, a specific model was proposed based on the functional protein analysis results. The healing of damaged cornea can be distinguished in 2 phases. Initially, CB-derivatives can act as immunomodulators of the inflammatory phase of the damaged cornea. Specifically, HGF, IDO, and PGE2 can induce the upstream activation of several protein family such as SEMAPHORIN and PLEXIN, efficiently regulating the overstimulated immune responses [69]. SEMAPHORINS and PLEXINS represents highly conserved protein families that have a crucial role to the neuronal axon guidance [70]. Recently, evidence has been provided regarding the new role of SEMAPHORINS and PLEXINS in an immune system. Specifically, SEMAPHORINS can modulate the over-stimulated immune responses. It has been demonstrated that both SEMAPHORINS and PLEXINS can induce T and B cell suppression [70]. Moreover, a series of events could follow, such as macrophage polarization (from M1 to M2) and activation of tolerogenic DCs. In parallel, M2 macrophage can further promote the tissue remodeling process, as has been reported in literature [42,43,44,45,46,47,48,49,50,51,52]. In the second phase, the growth factors of CB-PL induce the cell migration, proliferation, and production of tissue remodeling proteins such as collagen, syndecan, and perlecan, as has been described previously [66], promoting the healing process of the damaged cornea. Additionally, the CB-PPP can be used to produce of eye drops, considering they contain several biomolecules which may have a beneficial effect on immune regulation and tissue healing of the damaged cornea. Finally, it should be mentioned that all CB-derivatives used for the production of eye drops with the potential of human application must be negative for infectious disease, based on the guidelines of the European pharmacopoeia [71] The detection of the presence of specific viruses (HIV I/II, HBV, CMV, HCV, HAV, WNV, T.cruzi, and microorganisms may require additional time and cost to the production process. However, the monitoring of infectious diseases remains the only way to avoid any contamination during the application of CB-derived eye drops to patients.

Furthermore, the limitations of this study are appropriate to be discussed. In this study, CECs derived from Wistar rats were used for the performance of the experimental conditions. Rat-derived CECs offer an available source of cells in order to assess the proliferation, migration, and wound healing potential when CB-PL and CB-PPP are applied. However, further experimental procedures using CECs of human origin should be performed in order to obtain safer conclusions regarding the clinical utility of the CB derivatives. Obtaining CECs of human origin is a demanding task, requiring the appropriate documentation to be prepared and informed consent from the patients to be acquired to obtain tissue biopsies for CECs isolation purposes. Conversely, animal-derived CECs represent an easy-to-access source of cells, share common features with the human-derived cells, thus a series of different experimental procedures can be applied. Once all data have been gathered and a specific mechanism of action focused on the beneficial properties of CB-derivatives has been proposed, then a greater validation study can be performed using CECs of human origin.

## 5. Conclusions

The results of this study demonstrated promising evidence for the potential use of CB- derivatives as eye-drops and their utilization in ophthalmology applications such as DES. Typically, the treatment involves the use of 4–6 applications (3 drops for each application) per day, thus requiring 12–18 drops/day for both eyes [72]. This can be translated to 480–720 μL per day, meaning that the obtained volumes of CB-PL (10.4 ± 1.1 mL) and CB-PPP (20.6 ± 2.7) can last over 2 and 4 weeks, respectively. Currently, specific guidelines to produce eye drops from the CBUs have been published [72]. In those directions, it is suggested that the concentration of PLTs, WBCs, and RBCs must be lower than 15 × 10^3^/μL, 0.5 × 10^6^/μL and 0.01 × 10^9^ /μL, with proven complete negative sterility tests. In our study, we have successfully produced CB-PL and CB-PPP free of any cellular materials (PLTs, WBCs, and RBCs), therefore these products might be considered as ideal candidates for ophthalmological applications such as DES. The fact that CB-PPP is characterized by low protein content can efficiently support the in vitro culture of CECs and used as a biologically derived eye drops for DES applications.

The data of this study may support the consideration and development of further studies, elucidating the therapeutic use of blood derivatives, manufactured by fact-accredited public cord blood banks. Considering our data, preliminary animal studies, pilot studies, or clinical trials must be established to ensure the safety and feasibility of CB-PL and CB-PPP application, which can lead in obtaining the appropriate regulatory approval for clinical use.

## Figures and Tables

**Table 1:** List of used primers.

Gene	Forward Primer	Reverse Primer	Size
*Collagen 1 A1 (Col1A1)*	GCTCCTCTTAGGGGCCACT	GCTCCTCTTAGGGGCCACT	91
*Syndecan type-1*	TCAGGGGATGACGACTCGTTT	CTCCTGCTCGAAGTAGCCAGA	81
*Perlecan*	TTCCAGATGGTCTATTTCCGGG	CTTGGCACTTGCATCCTCC	84
*b-actin*	AGCTTCGGCACATATTTCATCTG	CGTTCACTCCCATGACAAACA	89

**Table 2:** Characteristics of CBUs, CB-PC, and CB-PPP.

*n* = 25	CBUs	CB-PC	CB-PPP	*p*-Value
Net Volume (mL)	139.4 ± 5.8	10.4 ± 1.1	20.7 ± 2.6	<0.001
WBCs (×10^3^/μL)	9.2 ± 1.9	1.3 ± 0.6	0.23 ± 0.2	<0.001
RBCs (×10^6^/μL)	3.6 ± 1.1	<0.1	<0.1	<0.001
HgB (g/dL)	11.3 ± 1.1	<0.1	<0.1	<0.001
PLTs (×10^3^/μL)	202.2 ± 27.9	1120.3 ± 70.4	16.1 ± 7.2	<0.001
PLTs (×10^9^)	28.1 ± 3.9	12.1 ± 1.5	0.3 ± 0.1	<0.001
PLTs Recovery (%)	-	42.3 ± 8.2	1.1 ± 0.3	<0.001

Statistically significant differences for the above parameters were observed between all groups (*p* < 0.001).

**Table 3:** Quality properties of CBUs, CB-PL, and CB-PPP.

(*n* = 25)	CBUs	CB-PL	CB-PPP	*p*-Value
pH	7.0 ± 0.7	7.1 ± 0.4	7.2 ± 0.6	NS
Lactate (mmol/l)	2.0 ± 0.7	2.1 ± 0.5	2.1 ± 0.4	NS
Glucose (mg/ dl)	354.5 ± 76.3	369.5 ± 83.3	355.7 ± 79.8	NS
PCO_2_ (mm Hg)	30.1 ± 2.2	31.4 ± 2.3	32.3 ± 3.1	NS
PO_2_ (mm Hg)	32.2 ± 2.4	30.5 ± 1.2	31.8 ± 3.2	NS
PT (sec)	14.2 ± 1.4	13.7 ± 3.2	14.7 ± 2.4	NS
APTT (sec)	34.3 ± 2.4	32.1 ± 4.1	34.3 ± 3.9	NS
Fibrinogen (mg/mL)	150.2 ± 15.2	151.3 ± 13.3	151.4 ± 12.1	NS

No statistically significant differences for the reported values were observed between all groups.

## Data Availability

Non applicable.

## Supplementary Materials

The following are available online at https://www.mdpi.com/article/10.3390/cimb44100303/s1, Table S1. Acceptable range of results outlined by the Hellenic Cord Blood Bank for processing and storage of cord blood units., Table S2. Sterility test of CBUs, CB-PL and CB-PPP, Figure S1. Percentage of CD marker expression of CECs from different groups and Table S3. Raw data file of proteins interactions using the STRING functional analysis tool.
