# Supplementary material for: Evaluation of the Regenerative Potential of Platelet-Lysate and Platelet-Poor Plasma Derived from the Cord Blood Units in Corneal Wound Healing Applications: An In Vitro Comparative Study on Corneal Epithelial Cells
Source: Curr Issues Mol Biol. 2022 Sep 22;44(10):4415–38. doi: 10.3390/cimb44100303 (PMC9600746; doi:10.3390/cimb44100303)
Supplement: Supplementary file 1 [file cimb-44-00303-s001.zip › cimb-1929337-supplementary.pdf]

Table S1. Acceptable range of results outlined by the Hellenic Cord Blood Bank for processing and storage of cord blood units.

Criteria	Unrelated	Related Allogeneic
**Collection bag mixed**	120 – 280 gr	100 – 220 gr
**WBC absolute count**	120 – 250 x 10^7 cells	100 – 250 x 10^7 cells
**WBC concentration**	13 – 29.5 cells/μL	10 – 29.5 cells/μL
**Cell Viability**	85 – 100%	85 – 100%

Table S2. Sterility test of CBUs, CB-PL and CB-PPP.

Tests	CBUs	CB-PL	CB-PPP	Acceptable Values
**Anti-HIV I/II**	Negative	Negative	Negative	<1.0 S/CO
**Anti-HBs**	Negative	Negative	Negative	<10 mIU/ml
**HBsAg**	Negative	Negative	Negative	<1.0 S/CO
**Anti-HBcore**	Negative	Negative	Negative	<1.0 S/CO
**Anti-HGV**	Negative	Negative	Negative	<1.0 S/CO
**Anti-HTLV I/II**	Negative	Negative	Negative	<1.0 S/CO
**Anti-CMV-IgG**	Negative	Negative	Negative	<1.0 AU/ml
**Anti-HCV**	Negative	Negative	Negative	<1.0 S/CO
**Anti-HAV**	Negative	Negative	Negative	<1.0 S/CO
**Anti-WNV-IgG**	Negative	Negative	Negative	<1.3 S/CO
**Anti-WNV-IgM**	Negative	Negative	Negative	<0.9 AU/ml
**Anti-T.cruzi**	Negative	Negative	Negative	<1.0 AU/ml
**BacT/Alert aerobic**	Negative	Negative	Negative	-

**BacT/Alert anaerobic**	Negative	Negative	Negative	-
**BacT/ Alert Myco**	Negative	Negative	Negative	-

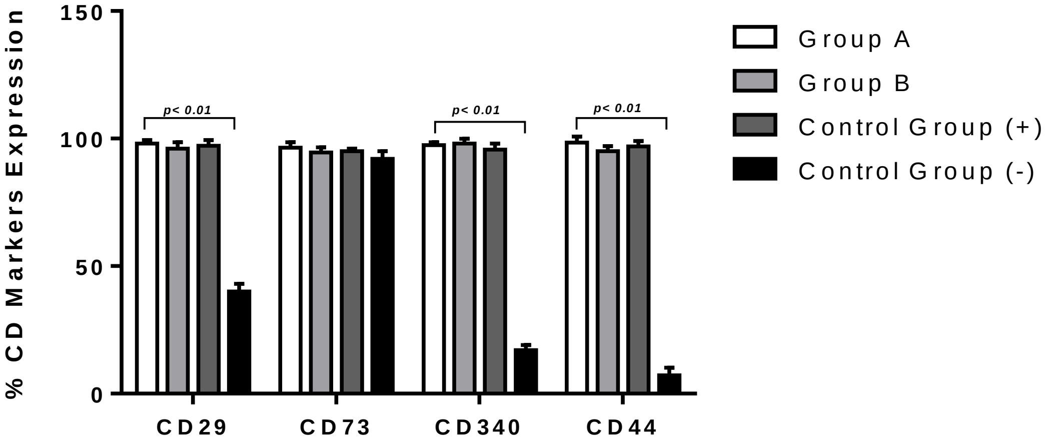

Figure S1. Percentage of CD marker expression of CECs from different groups. CECs from groups A, B and positive control group presented > 95 % expression for the markers CD29, CD73, CD340 and CD44. On the other hand, CECs of negative control group presented lower expression of CD29, CD340 and CD44 compared to the above groups. Non parametric Kruskal Wallis test showed statistically significant differences regarding the % CD markers expression between the negative control group and the rest of them ($p< 0.001$). Group A: CECs cultured with α-MEM supplemented with 20% v/v CB-PL), Group B: CECs cultured with α-MEM supplemented with 20% v/v CB-PPP, Control group (+): Positive control group, CECs cultured with complete culture me-dium, Control group (-), negative control group: CECs cultured only with α-MEM.

Table S3. Raw data file of proteins interactions using the STRING functional analysis tool.

```
#node identifier x_position y_position color annotation
ACVRL1     9606.ENSP00000373574  0.8245971563981044
      0.3787200832466181    rgb(255,255,255) Serine/threonine-
protein kinase receptor R3; Type I receptor for TGF-beta
family ligands BMP9/GDF2 and BMP10 and important regulator of
normal blood vessel development. On ligand binding, forms a
receptor complex consisting of two type II and two type I
transmembrane serine/threonine kinases. Type II receptors
phosphorylate and activate type I receptors which
autophosphorylate, then bind and activate SMAD
transcriptional regulators. May bind activin as well; Belongs
to the protein kinase superfamily. TKL Ser/Thr protein kinase
family. TGFB receptor subfamily
ANGPT2     9606.ENSP00000314897  0.5959715639810428
      0.7477107180020812    rgb(255,255,255) Angiopoietin-2;
Binds to TEK/TIE2, competing for the ANGPT1 binding site, and
modulating ANGPT1 signaling. Can induce tyrosine
phosphorylation of TEK/TIE2 in the absence of ANGPT1. In the
absence of angiogenic inducers, such as VEGF, ANGPT2-mediated
loosening of cell-matrix contacts may induce endothelial cell
apoptosis with consequent vascular regression. In concert
with VEGF, it may facilitate endothelial cell migration and
proliferation, thus serving as a permissive angiogenic
signal; Fibrinogen C domain containing
CAV1  9606.ENSP00000339191  0.5209004739336492
      0.3131633714880333    rgb(255,255,255) Caveolin-1; May
act as a scaffolding protein within caveolar membranes.
Interacts directly with G-protein alpha subunits and can
functionally regulate their activity (By similarity).
Involved in the costimulatory signal essential for T-cell
receptor (TCR)- mediated T-cell activation. Its binding to
DPP4 induces T-cell proliferation and NF-kappa-B activation
in a T-cell receptor/CD3- dependent manner. Recruits CTNNB1
to caveolar membranes and may regulate CTNNB1-mediated
signaling through the Wnt pathway. Negatively regulates
TGFB1-mediated activation of SMAD2/3 by mediating the interna
[...]
CBL   9606.ENSP00000264033  0.2334123222748815
      0.4751821019771072    rgb(255,255,255) E3 ubiquitin-
protein ligase CBL; Adapter protein that functions as a
```

negative regulator of many signaling pathways that are triggered by activation of cell surface receptors. Acts as an E3 ubiquitin-protein ligase, which accepts ubiquitin from specific E2 ubiquitin-conjugating enzymes, and then transfers it to substrates promoting their degradation by the proteasome. Recognizes activated receptor tyrosine kinases, including KIT, FLT1, FGFR1, FGFR2, PDGFRA, PDGFRB, EGFR, CSF1R, EPHA8 and KDR and terminates signaling. Recognizes membrane-bound HCK, SRC and other kinases of the SRC family [...]

CD44 9606.ENSP00000398632 0.4936018957345971 0.4058792924037461 rgb(255,255,255) CD44 antigen; Receptor for hyaluronic acid (HA). Mediates cell-cell and cell-matrix interactions through its affinity for HA, and possibly also through its affinity for other ligands such as osteopontin, collagens, and matrix metalloproteinases (MMPs). Adhesion with HA plays an important role in cell migration, tumor growth and progression. In cancer cells, may play an important role in invadopodia formation. Also involved in lymphocyte activation, recirculation and homing, and in hematopoiesis. Altered expression or dysfunction causes numerous pathogenic phenotypes. Great protein hete [...]

CDH1 9606.ENSP00000261769 0.46545023696682464 0.3187825182101977 rgb(255,255,255) Cadherin-1; Cadherins are calcium-dependent cell adhesion proteins. They preferentially interact with themselves in a homophilic manner in connecting cells; cadherins may thus contribute to the sorting of heterogeneous cell types. CDH1 is involved in mechanisms regulating cell-cell adhesions, mobility and proliferation of epithelial cells. Has a potent invasive suppressor role. It is a ligand for integrin alpha-E/beta-7

CDH5 9606.ENSP00000344115 0.5695260663507109 0.4648803329864724 rgb(255,255,255) Cadherin-5; Cadherins are calcium-dependent cell adhesion proteins. They preferentially interact with themselves in a homophilic manner in connecting cells; cadherins may thus contribute to the sorting of heterogeneous cell types. This cadherin may play a important role in endothelial cell biology through control of the cohesion and organization of the intercellular junctions. It associates with alpha-catenin forming a link to the cytoskeleton. Acts in concert with KRIT1 to establish and maintain correct endothelial cell polarity and vascular lumen. These effects are mediated by recrui [...]

CTNNB1 9606.ENSP00000344456 0.5046919431279621 0.35811654526534864 rgb(255,255,255) Catenin beta-1; Key downstream component of the canonical Wnt signaling pathway. In the absence of Wnt, forms a complex with AXIN1, AXIN2, APC, CSNK1A1 and GSK3B that promotes phosphorylation on N-terminal Ser and Thr residues and ubiquitination of CTNNB1 via BTRC and its subsequent degradation by the proteasome. In the presence of Wnt ligand, CTNNB1 is not ubiquitinated and accumulates in the nucleus, where it acts as a coactivator for transcription factors of the TCF/LEF family, leading to activate Wnt responsive genes. Involved in the regulation of cell adhesion, as component of an [...]

```
DCN   9606.ENSP00000052754  0.7921800947867299
      0.4882934443288241    rgb(255,255,255) Decorin; May
affect the rate of fibrils formation; Small leucine rich
repeat proteoglycans
ENG   9606.ENSP00000362299  0.7281990521327015
      0.4714360041623309    rgb(239,157,117) Endoglin;
Vascular endothelium glycoprotein that plays an important
role in the regulation of angiogenesis. Required for normal
structure and integrity of adult vasculature. Regulates the
migration of vascular endothelial cells. Required for normal
extraembryonic angiogenesis and for embryonic heart
development (By similarity). May play a critical role in the
binding of endothelial cells to integrins and/or other RGD
receptors. Acts as TGF-beta coreceptor and is involved in the
TGF-beta/BMP signaling cascade that ultimately leads to the
activation of SMAD transcription factors. Require [...]
FGF1  9606.ENSP00000480791  0.32895734597156395
      0.6746618106139439    rgb(101,199,255) Fibroblast growth
factor 1; Plays an important role in the regulation of cell
survival, cell division, angiogenesis, cell differentiation
and cell migration. Functions as potent mitogen in vitro.
Acts as a ligand for FGFR1 and integrins. Binds to FGFR1 in
the presence of heparin leading to FGFR1 dimerization and
activation via sequential autophosphorylation on tyrosine
residues which act as docking sites for interacting proteins,
leading to the activation of several signaling cascades.
Binds to integrin ITGAV:ITGB3. Its binding to integrin,
subsequent ternary complex formation with int [...]
FGF2  9606.ENSP00000264498  0.484218009478673
      0.5163891779396462    rgb(255,255,255) Fibroblast growth
factor 2; Plays an important role in the regulation of cell
survival, cell division, angiogenesis, cell differentiation
and cell migration. Functions as potent mitogen in vitro. Can
induce angiogenesis; Belongs to the heparin-binding growth
factors family
FGFBP1     9606.ENSP00000371770  0.08753554502369669
      0.527627471383975     rgb(255,255,255) Fibroblast growth
factor-binding protein 1; Acts as a carrier protein that
release fibroblast- binding factors (FGFs) from the
extracellular matrix (EM) storage and thus enhance the
mitogenic activity of FGFs. Enhances FGF2 signaling during
tissue repair, angiogenesis and in tumor growth
FGFR1 9606.ENSP00000393312  0.41682464454976303
      0.566024973985432     rgb(255,255,255) Fibroblast growth
factor receptor 1; Tyrosine-protein kinase that acts as cell-
surface receptor for fibroblast growth factors and plays an
essential role in the regulation of embryonic development,
cell proliferation, differentiation and migration. Required
for normal mesoderm patterning and correct axial organization
during embryonic development, normal skeletogenesis and
normal development of the gonadotropin-releasing hormone
(GnRH) neuronal system. Phosphorylates PLCG1, FRS2, GAB1 and
SHB. Ligand binding leads to the activation of several
signaling cascades. Activation of PLCG1 lea [...]
FGFR2 9606.ENSP00000410294  0.29995260663507106
```

0.4704994797086368 rgb(255,255,255) Fibroblast growth factor receptor 2; Tyrosine-protein kinase that acts as cell-surface receptor for fibroblast growth factors and plays an essential role in the regulation of cell proliferation, differentiation, migration and apoptosis, and in the regulation of embryonic development. Required for normal embryonic patterning, trophoblast function, limb bud development, lung morphogenesis, osteogenesis and skin development. Plays an essential role in the regulation of osteoblast differentiation, proliferation and apoptosis, and is required for normal skeleton development. Promotes cell p [...]
FGFR3 9606.ENSP00000339824 0.33407582938388625
0.4105619146722165 rgb(255,255,255) Fibroblast growth factor receptor 3; Tyrosine-protein kinase that acts as cell-surface receptor for fibroblast growth factors and plays an essential role in the regulation of cell proliferation, differentiation and apoptosis. Plays an essential role in the regulation of chondrocyte differentiation, proliferation and apoptosis, and is required for normal skeleton development. Regulates both osteogenesis and postnatal bone mineralization by osteoblasts. Promotes apoptosis in chondrocytes, but can also promote cancer cell proliferation. Required for normal development of the inner ear. Ph [...]
FGFR4 9606.ENSP00000292408 0.2828909952606635
0.4236732570239334 rgb(255,255,255) Fibroblast growth factor receptor 4; Tyrosine-protein kinase that acts as cell-surface receptor for fibroblast growth factors and plays a role in the regulation of cell proliferation, differentiation and migration, and in regulation of lipid metabolism, bile acid biosynthesis, glucose uptake, vitamin D metabolism and phosphate homeostasis. Required for normal down-regulation of the expression of CYP7A1, the rate-limiting enzyme in bile acid synthesis, in response to FGF19. Phosphorylates PLCG1 and FRS2. Ligand binding leads to the activation of several signaling cascades. Activation of [...]
FIGF 9606.ENSP00000297904 0.39208530805687203
0.7795525494276796 rgb(255,255,255) Vascular endothelial growth factor D; Growth factor active in angiogenesis, lymphangiogenesis and endothelial cell growth, stimulating their proliferation and migration and also has effects on the permeability of blood vessels. May function in the formation of the venous and lymphatic vascular systems during embryogenesis, and also in the maintenance of differentiated lymphatic endothelium in adults. Binds and activates VEGFR-2 (KDR/FLK1) and VEGFR-3 (FLT4) receptors
FKBP1A 9606.ENSP00000383003 0.7401421800947868
0.13803329864724245 rgb(255,255,255) Peptidyl-prolyl cis-trans isomerase FKBP1A; Keeps in an inactive conformation TGFBR1, the TGF-beta type I serine/threonine kinase receptor, preventing TGF-beta receptor activation in absence of ligand. Recruits SMAD7 to ACVR1B which prevents the association of SMAD2 and SMAD3 with the activin receptor complex, thereby blocking the activin signal. May modulate the RYR1 calcium channel activity. PPIases accelerate the folding of proteins.

It catalyzes the cis-trans isomerization of proline imidic peptide bonds in oligopeptides; Belongs to the FKBP-type PPIase family. FKBP1 subfamily
FLT1 9606.ENSP00000282397 0.5200473933649289 0.7083766909469303 rgb(239,130,117) Vascular endothelial growth factor receptor 1; Tyrosine-protein kinase that acts as a cell-surface receptor for VEGFA, VEGFB and PGF, and plays an essential role in the development of embryonic vasculature, the regulation of angiogenesis, cell survival, cell migration, macrophage function, chemotaxis, and cancer cell invasion. May play an essential role as a negative regulator of embryonic angiogenesis by inhibiting excessive proliferation of endothelial cells. Can promote endothelial cell proliferation, survival and angiogenesis in adulthood. Its function in promoting cell proliferati [...]
FLT4 9606.ENSP00000261937 0.4680094786729858 0.7973465140478668 rgb(150,101,255) Vascular endothelial growth factor receptor 3; Tyrosine-protein kinase that acts as a cell-surface receptor for VEGFC and VEGFD, and plays an essential role in adult lymphangiogenesis and in the development of the vascular network and the cardiovascular system during embryonic development. Promotes proliferation, survival and migration of endothelial cells, and regulates angiogenic sprouting. Signaling by activated FLT4 leads to enhanced production of VEGFC, and to a lesser degree VEGFA, thereby creating a positive feedback loop that enhances FLT4 signaling. Modulates KDR signaling by [...]
FN1 9606.ENSP00000346839 0.5754976303317536 0.5154526534859522 rgb(255,255,255) Fibronectin 1; Fibronectin type III domain containing; Endogenous ligands
GAB1 9606.ENSP00000262995 0.20014218009478674 0.5941207075962539 rgb(255,255,255) GRB2-associated-binding protein 1; Adapter protein that plays a role in intracellular signaling cascades triggered by activated receptor-type kinases. Plays a role in FGFR1 signaling. Probably involved in signaling by the epidermal growth factor receptor (EGFR) and the insulin receptor (INSR); Pleckstrin homology domain containing
GDF2 9606.ENSP00000463051 0.8049763033175356 0.2363683662851197 rgb(255,255,255) Growth/differentiation factor 2; Potent circulating inhibitor of angiogenesis. Signals through the type I activin receptor ACVRL1 but not other Alks. Signaling through SMAD1 in endothelial cells requires TGF-beta coreceptor endoglin/ENG
GIPC1 9606.ENSP00000376753 0.8100947867298578 0.6503121748178982 rgb(255,255,255) PDZ domain-containing protein GIPC1; May be involved in G protein-linked signaling; Belongs to the GIPC family
GNAI1 9606.ENSP00000343027 0.08668246445497631 0.42460978147762746 rgb(255,255,255) Guanine nucleotide-binding protein G(i) subunit alpha-1; Guanine nucleotide-binding proteins (G proteins) function as transducers downstream of G protein-coupled receptors (GPCRs) in numerous signaling cascades. The alpha chain contains the

guanine nucleotide binding site and alternates between an active, GTP-bound state and an inactive, GDP-bound state. Signaling by an activated GPCR promotes GDP release and GTP binding. The alpha subunit has a low GTPase activity that converts bound GTP to GDP, thereby terminating the signal. Both GDP release and GTP hydrolysis are modulated by numer [...]
GRB2 9606.ENSP00000376345 0.27777251184834123
0.5154526534859522 rgb(255,255,255) Growth factor receptor-bound protein 2; Adapter protein that provides a critical link between cell surface growth factor receptors and the Ras signaling pathway; SH2 domain containing
HGF 9606.ENSP00000222390 0.5277251184834124
0.6081685744016649 rgb(255,175,101) Hepatocyte growth factor; Potent mitogen for mature parenchymal hepatocyte cells, seems to be a hepatotrophic factor, and acts as a growth factor for a broad spectrum of tissues and cell types. Activating ligand for the receptor tyrosine kinase MET by binding to it and promoting its dimerization; Deafness associated genes
HIF1A 9606.ENSP00000437955 0.6582464454976303
0.5341831425598335 rgb(255,255,255) Hypoxia-inducible factor 1-alpha; Functions as a master transcriptional regulator of the adaptive response to hypoxia. Under hypoxic conditions, activates the transcription of over 40 genes, including erythropoietin, glucose transporters, glycolytic enzymes, vascular endothelial growth factor, HILPDA, and other genes whose protein products increase oxygen delivery or facilitate metabolic adaptation to hypoxia. Plays an essential role in embryonic vascularization, tumor angiogenesis and pathophysiology of ischemic disease. Binds to core DNA sequence 5'-[AG]CGTG-3' within the hypoxia res [...]
HSP90AA1 9606.ENSP00000335153 0.596824644549763
0.44053069719042665 rgb(255,255,255) Heat shock protein HSP 90-alpha; Molecular chaperone that promotes the maturation, structural maintenance and proper regulation of specific target proteins involved for instance in cell cycle control and signal transduction. Undergoes a functional cycle that is linked to its ATPase activity which is essential for its chaperone activity. This cycle probably induces conformational changes in the client proteins, thereby causing their activation. Interacts dynamically with various co-chaperones that modulate its substrate recognition, ATPase cycle and chaperone function. Engages with a ra [...]
IDO1 9606.ENSP00000430950 0.279478672985782
0.17736732570239333 rgb(101,255,163) Indoleamine 2,3-dioxygenase 1; Catalyzes the first and rate limiting step of the catabolism of the essential amino acid tryptophan along the kynurenine pathway. Involved in the peripheral immune tolerance, contributing to maintain homeostasis by preventing autoimmunity or immunopathology that would result from uncontrolled and overreacting immune responses. Tryptophan shortage inhibits T lymphocytes division and accumulation of tryptophan catabolites induces T-cell apoptosis and

differentiation of regulatory T-cells. Acts as a suppressor of anti-tumor immunity. Limits the growth of int [...]
IQGAP1 9606.ENSP00000268182 0.2922748815165877 0.27851196670135275 rgb(255,255,255) Iq motif containing gtpase activating protein 1; Ras GTPase-activating-like protein IQGAP1; Binds to activated CDC42 but does not stimulate its GTPase activity. It associates with calmodulin. Could serve as an assembly scaffold for the organization of a multimolecular complex that would interface incoming signals to the reorganization of the actin cytoskeleton at the plasma membrane. May promote neurite outgrowth
ITGAV 9606.ENSP00000261023 0.40488151658767774 0.2700832466181062 rgb(255,255,255) Integrin alpha-V; The alpha-V (ITGAV) integrins are receptors for vitronectin, cytotactin, fibronectin, fibrinogen, laminin, matrix metalloproteinase-2, osteopontin, osteomodulin, prothrombin, thrombospondin and vWF. They recognize the sequence R-G-D in a wide array of ligands. ITGAV:ITGB3 binds to fractalkine (CX3CL1) and may act as its coreceptor in CX3CR1-dependent fractalkine signaling. ITGAV:ITGB3 binds to NRG1 (via EGF domain) and this binding is essential for NRG1-ERBB signaling. ITGAV:ITGB3 binds to FGF1 and this binding is essential for FGF1 signaling. ITGAV:ITGB3 binds to IGF [...]
ITGB1 9606.ENSP00000379350 0.452654028436019 0.4892299687825182 rgb(255,255,255) Integrin beta-1; Integrins alpha-1/beta-1, alpha-2/beta-1, alpha-10/beta- 1 and alpha-11/beta-1 are receptors for collagen. Integrins alpha- 1/beta-1 and alpha-2/beta-2 recognize the proline-hydroxylated sequence G-F-P-G-E-R in collagen. Integrins alpha-2/beta-1, alpha- 3/beta-1, alpha-4/beta-1, alpha-5/beta-1, alpha-8/beta-1, alpha- 10/beta-1, alpha-11/beta-1 and alpha-V/beta-1 are receptors for fibronectin. Alpha-4/beta-1 recognizes one or more domains within the alternatively spliced CS-1 and CS-5 regions of fibronectin. Integrin alpha-5/beta-1 is a receptor for fibrinogen. Integrin [...]
ITGB3 9606.ENSP00000452786 0.3622274881516587 0.34219562955254945 rgb(255,255,255) Integrin beta-3; Integrin alpha-V/beta-3 (ITGAV:ITGB3) is a receptor for cytotactin, fibronectin, laminin, matrix metalloproteinase-2, osteopontin, osteomodulin, prothrombin, thrombospondin, vitronectin and von Willebrand factor. Integrin alpha-IIb/beta-3 (ITGA2B:ITGB3) is a receptor for fibronectin, fibrinogen, plasminogen, prothrombin, thrombospondin and vitronectin. Integrins alpha-IIb/beta-3 and alpha-V/beta-3 recognize the sequence R-G-D in a wide array of ligands. Integrin alpha- IIb/beta-3 recognizes the sequence H-H-L-G-G-G-A-K-Q-A-G-D-V in fibrinogen gamma chain. Following act [...]
KDR 9606.ENSP00000263923 0.5618483412322275 0.6362643080124871 rgb(239,117,117) Vascular endothelial growth factor receptor 2; Tyrosine-protein kinase that acts as a cell-surface receptor for VEGFA, VEGFC and VEGFD. Plays an essential role in the regulation of

angiogenesis, vascular development, vascular permeability, and embryonic hematopoiesis. Promotes proliferation, survival, migration and differentiation of endothelial cells. Promotes reorganization of the actin cytoskeleton. Isoforms lacking a transmembrane domain, such as isoform 2 and isoform 3, may function as decoy receptors for VEGFA, VEGFC and/or VEGFD. Isoform 2 plays an important role as negative reg [...]

LTBP1 9606.ENSP00000386043 0.8834597156398105 0.27851196670135275 rgb(255,255,255) Latent-transforming growth factor beta-binding protein 1; May be involved in the assembly, secretion and targeting of TGFB1 to sites at which it is stored and/or activated. May play critical roles in controlling and directing the activity of TGFB1. May have a structural role in the extracellular matrix (ECM); Latent transforming growth factor beta binding proteins

MET 9606.ENSP00000317272 0.5106635071090048 0.6578043704474507 rgb(239,137,117) Hepatocyte growth factor receptor; Receptor tyrosine kinase that transduces signals from the extracellular matrix into the cytoplasm by binding to hepatocyte growth factor/HGF ligand. Regulates many physiological processes including proliferation, scattering, morphogenesis and survival. Ligand binding at the cell surface induces autophosphorylation of MET on its intracellular domain that provides docking sites for downstream signaling molecules. Following activation by ligand, interacts with the PI3-kinase subunit PIK3R1, PLCG1, SRC, GRB2, STAT3 or the adapter GAB1. Recruitment of thes [...]

MMP9 9606.ENSP00000361405 0.6531279620853081 0.45551508844953176 rgb(255,255,255) Matrix metalloproteinase-9; May play an essential role in local proteolysis of the extracellular matrix and in leukocyte migration. Could play a role in bone osteoclastic resorption. Cleaves KiSS1 at a Gly-|-Leu bond. Cleaves type IV and type V collagen into large C-terminal three quarter fragments and shorter N-terminal one quarter fragments. Degrades fibronectin but not laminin or Pz-peptide; M10 matrix metallopeptidases

NCK1 9606.ENSP00000417273 0.2675355450236967 0.7477107180020812 rgb(255,255,255) Cytoplasmic protein NCK1; Adapter protein which associates with tyrosine-phosphorylated growth factor receptors, such as KDR and PDGFRB, or their cellular substrates. Maintains low levels of EIF2S1 phosphorylation by promoting its dephosphorylation by PP1. Plays a role in the DNA damage response, not in the detection of the damage by ATM/ATR, but for efficient activation of downstream effectors, such as that of CHEK2. Plays a role in ELK1-dependent transcriptional activation in response to activated Ras signaling. Modulates the activation of EIF2AK2/PKR by dsRNA. May play a role in ce [...]

NCK2 9606.ENSP00000233154 0.26412322274881517 0.7973465140478668 rgb(255,255,255) Cytoplasmic protein NCK2; Adapter protein which associates with tyrosine-

phosphorylated growth factor receptors or their cellular substrates. Maintains low levels of EIF2S1 phosphorylation by promoting its dephosphorylation by PP1. Plays a role in ELK1-dependent transcriptional activation in response to activated Ras signaling; SH2 domain containing
NRP1 9606.ENSP00000265371 0.6923696682464456 0.6559313215400625 rgb(239,123,117) Neuropilin-1; The membrane-bound isoform 1 is a receptor involved in the development of the cardiovascular system, in angiogenesis, in the formation of certain neuronal circuits and in organogenesis outside the nervous system. It mediates the chemorepulsant activity of semaphorins. It binds to semaphorin 3A, The PLGF-2 isoform of PGF, The VEGF165 isoform of VEGFA and VEGFB. Coexpression with KDR results in increased VEGF165 binding to KDR as well as increased chemotaxis. Regulate VEGF-induced angiogenesis. Binding to VEGFA initiates a signaling pathway needed for motor neuron axon guid [...]
NRP2 9606.ENSP00000353582 0.706872037914692 0.8226326742976068 rgb(239,150,117) Neuropilin-2; High affinity receptor for semaphorins 3C, 3F, VEGF-165 and VEGF-145 isoforms of VEGF, and the PLGF-2 isoform of PGF
NTRK1 9606.ENSP00000431418 0.4475355450236967 0.6821540062434964 rgb(255,255,255) High affinity nerve growth factor receptor; Receptor tyrosine kinase involved in the development and the maturation of the central and peripheral nervous systems through regulation of proliferation, differentiation and survival of sympathetic and nervous neurons. High affinity receptor for NGF which is its primary ligand. Can also bind and be activated by NTF3/neurotrophin-3. However, NTF3 only supports axonal extension through NTRK1 but has no effect on neuron survival (By similarity). Upon dimeric NGF ligand-binding, undergoes homodimerization, autophosphorylation and activation. Rec [...]
PDGFA 9606.ENSP00000346508 0.2965402843601896 0.6418834547346515 rgb(178,171,0) Platelet-derived growth factor subunit A; Growth factor that plays an essential role in the regulation of embryonic development, cell proliferation, cell migration, survival and chemotaxis. Potent mitogen for cells of mesenchymal origin. Required for normal lung alveolar septum formation during embryogenesis, normal development of the gastrointestinal tract, normal development of Leydig cells and spermatogenesis. Required for normal oligodendrocyte development and normal myelination in the spinal cord and cerebellum. Plays an important role in wound healing. Signaling is modulated by t [...]
PDGFB 9606.ENSP00000330382 0.3724644549763033 0.6259625390218523 rgb(255,255,255) Platelet-derived growth factor subunit B; Growth factor that plays an essential role in the regulation of embryonic development, cell proliferation, cell migration, survival and chemotaxis. Potent mitogen for cells of mesenchymal origin. Required for normal proliferation and recruitment of pericytes and vascular smooth muscle cells in the central nervous system,

skin, lung, heart and placenta. Required for normal blood vessel development, and for normal development of kidney glomeruli. Plays an important role in wound healing. Signaling is modulated by the formation of heterodimers wit [...]
PDGFC 9606.ENSP00000422464 0.31274881516587677 0.7814255983350678 rgb(255,255,255) Platelet-derived growth factor C; Growth factor that plays an essential role in the regulation of embryonic development, cell proliferation, cell migration, survival and chemotaxis. Potent mitogen and chemoattractant for cells of mesenchymal origin. Required for normal skeleton formation during embryonic development, especially for normal development of the craniofacial skeleton and for normal development of the palate. Required for normal skin morphogenesis during embryonic development. Plays an important role in wound healing, where it appears to be involved in three stages: inflamma [...]
PDGFRA 9606.ENSP00000257290 0.38355450236966826 0.5145161290322581 rgb(0,28,178) Platelet-derived growth factor receptor alpha; Tyrosine-protein kinase that acts as a cell-surface receptor for PDGFA, PDGFB and PDGFC and plays an essential role in the regulation of embryonic development, cell proliferation, survival and chemotaxis. Depending on the context, promotes or inhibits cell proliferation and cell migration. Plays an important role in the differentiation of bone marrow-derived mesenchymal stem cells. Required for normal skeleton development and cephalic closure during embryonic development. Required for normal development of the mucosa lining the gastrointes [...]
PDGFRB 9606.ENSP00000261799 0.4338862559241706 0.6100416233090531 rgb(255,255,255) Platelet-derived growth factor receptor beta; Tyrosine-protein kinase that acts as cell-surface receptor for homodimeric PDGFB and PDGFD and for heterodimers formed by PDGFA and PDGFB, and plays an essential role in the regulation of embryonic development, cell proliferation, survival, differentiation, chemotaxis and migration. Plays an essential role in blood vessel development by promoting proliferation, migration and recruitment of pericytes and smooth muscle cells to endothelial cells. Plays a role in the migration of vascular smooth muscle cells and the formation of neointima at v [...]
PECAM1 9606.ENSP00000457421 0.6232701421800949 0.5988033298647243 rgb(255,255,255) Platelet endothelial cell adhesion molecule; Cell adhesion molecule which is required for leukocyte transendothelial migration (TEM) under most inflammatory conditions. Tyr-690 plays a critical role in TEM and is required for efficient trafficking of PECAM1 to and from the lateral border recycling compartment (LBRC) and is also essential for the LBRC membrane to be targeted around migrating leukocytes. Heterophilic interaction with CD177 plays a role in transendothelial migration of neutrophils. Homophilic ligation of PECAM1 prevents macrophage-mediated phagocytosis of neighboring viab [...]

PGF 9606.ENSP00000451040 0.5362559241706162
0.7926638917793966 rgb(255,255,255) Placental growth factor; Placenta growth factor; Growth factor active in angiogenesis and endothelial cell growth, stimulating their proliferation and migration. It binds to the receptor FLT1/VEGFR-1. Isoform PlGF-2 binds NRP1/neuropilin-1 and NRP2/neuropilin-2 in a heparin-dependent manner. Also promotes cell tumor growth
PLXNA1 9606.ENSP00000377061 0.801563981042654
0.7645681581685744 rgb(255,255,255) Plexin-A1; Coreceptor for SEMA3A, SEMA3C, SEMA3F and SEMA6D. Necessary for signaling by class 3 semaphorins and subsequent remodeling of the cytoskeleton. Plays a role in axon guidance, invasive growth and cell migration. Class 3 semaphorins bind to a complex composed of a neuropilin and a plexin. The plexin modulates the affinity of the complex for specific semaphorins, and its cytoplasmic domain is required for the activation of down- stream signaling events in the cytoplasm (By similarity)
PLXNA2 9606.ENSP00000356000 0.8450710900473934
0.8619667013527577 rgb(255,255,255) Plexin-A2; Coreceptor for SEMA3A and SEMA6A. Necessary for signaling by SEMA6A and class 3 semaphorins and subsequent remodeling of the cytoskeleton. Plays a role in axon guidance, invasive growth and cell migration. Class 3 semaphorins bind to a complex composed of a neuropilin and a plexin. The plexin modulates the affinity of the complex for specific semaphorins, and its cytoplasmic domain is required for the activation of down- stream signaling events in the cytoplasm (By similarity)
PLXNA4 9606.ENSP00000352882 0.8356872037914693
0.7261706555671176 rgb(255,255,255) Plexin-A4; Coreceptor for SEMA3A. Necessary for signaling by class 3 semaphorins and subsequent remodeling of the cytoskeleton. Plays a role in axon guidance in the developing nervous system. Class 3 semaphorins bind to a complex composed of a neuropilin and a plexin. The plexin modulates the affinity of the complex for specific semaphorins, and its cytoplasmic domain is required for the activation of down-stream signaling events in the cytoplasm (By similarity)
PLXNC1 9606.ENSP00000258526 0.8646919431279622
0.7926638917793966 rgb(255,255,255) Plexin-C1; Receptor for SEMA7A, for smallpox semaphorin A39R, vaccinia virus semaphorin A39R and for herpesvirus Sema protein. Binding of semaphorins triggers cellular responses leading to the rearrangement of the cytoskeleton and to secretion of IL6 and IL8 (By similarity); Belongs to the plexin family
PLXND1 9606.ENSP00000317128 0.7529383886255925
0.907856399583767 rgb(255,255,255) Plexin-D1; Cell surface receptor for SEMA4A and for class 3 semaphorins, such as SEMA3A, SEMA3C and SEMA3E. Plays an important role in cell-cell signaling, and in regulating the migration of a wide spectrum of cell types. Regulates the migration of thymocytes in the medulla. Regulates endothelial cell migration. Plays an important role in ensuring the

specificity of synapse formation. Required for normal development of the heart and vasculature (By similarity). Mediates anti-angiogenic signaling in response to SEMA3E; Belongs to the plexin family
PTGR1 9606.ENSP00000385763 0.9073459715639811 0.09682622268470344 rgb(187,255,101) Prostaglandin reductase 1; Functions as 15-oxo-prostaglandin 13-reductase and acts on 15-oxo-PGE1, 15-oxo-PGE2 and 15-oxo-PGE2-alpha. Has no activity towards PGE1, PGE2 and PGE2-alpha (By similarity). Catalyzes the conversion of leukotriene B4 into its biologically less active metabolite, 12-oxo-leukotriene B4. This is an initial and key step of metabolic inactivation of leukotriene B4
RGS14 9606.ENSP00000386229 0.12165876777251186 0.3337669094693028 rgb(14,178,0) Regulator of G-protein signaling 14; Regulates G protein-coupled receptor signaling cascades. Inhibits signal transduction by increasing the GTPase activity of G protein alpha subunits, thereby driving them into their inactive GDP-bound form. Besides, modulates signal transduction via G protein alpha subunits by functioning as a GDP-dissociation inhibitor (GDI). Has GDI activity on G(i) alpha subunits GNAI1 and GNAI3, but not on GNAI2 and G(o) alpha subunit GNAO1. Has GAP activity on GNAI0, GNAI2 and GNAI3. May act as a scaffold integrating G protein and Ras/Raf MAPkinase signaling pat [...]
S100A13 9606.ENSP00000357688 0.12933649289099525 0.8338709677419356 rgb(255,255,255) Protein S100-A13; Plays a role in the export of proteins that lack a signal peptide and are secreted by an alternative pathway. Binds two calcium ions per subunit. Binds one copper ion. Binding of one copper ion does not interfere with calcium binding. Required for the copper-dependent stress-induced export of IL1A and FGF1. The calcium-free protein binds to lipid vesicles containing phosphatidylserine, but not to vesicles containing phosphatidylcholine (By similarity)
SDC1 9606.ENSP00000370542 0.6898104265402845 0.5145161290322581 rgb(255,255,255) Syndecan-1; Cell surface proteoglycan that bears both heparan sulfate and chondroitin sulfate and that links the cytoskeleton to the interstitial matrix. Regulates exosome biogenesis in concert with SDCBP and PDCD6IP
SEMA3A 9606.ENSP00000265362 0.6761611374407583 0.7870447450572322 rgb(255,255,255) Semaphorin-3A; Involved in the development of the olfactory system and in neuronal control of puberty. Induces the collapse and paralysis of neuronal growth cones. Could serve as a ligand that guides specific growth cones by a motility-inhibiting mechanism. Binds to the complex neuropilin-1/plexin-1; Immunoglobulin like domain containing
SEMA3B 9606.ENSP00000484146 0.6556872037914692 0.9031737773152967 rgb(255,255,255) Semaphorin-3B; Inhibits axonal extension by providing local signals to specify territories inaccessible for growing axons; Belongs to the semaphorin family

SEMA3C 9606.ENSP00000265361 0.682132701421801 0.8966181061394382 rgb(255,255,255) Semaphorin-3C; Binds to plexin family members and plays an important role in the regulation of developmental processes. Required for normal cardiovascular development during embryogenesis. Functions as attractant for growing axons, and thereby plays an important role in axon growth and axon guidance (By similarity); I-set domain containing
SEMA3D 9606.ENSP00000284136 0.7623222748815166 0.8198231009365246 rgb(255,255,255) Semaphorin-3D; Induces the collapse and paralysis of neuronal growth cones. Could potentially act as repulsive cues toward specific neuronal populations. Binds to neuropilin (By similarity); Semaphorins
SEMA3F 9606.ENSP00000002829 0.8501895734597157 0.6746618106139439 rgb(255,255,255) Semaphorin-3F; May play a role in cell motility and cell adhesion; Immunoglobulin like domain containing
SH2D2A 9606.ENSP00000376123 0.4210900473933649 0.8413631633714881 rgb(255,255,255) SH2 domain-containing protein 2A; Could be a T-cell-specific adapter protein involved in the control of T-cell activation. May play a role in the CD4-p56- LCK-dependent signal transduction pathway. Could also play an important role in normal and pathological angiogenesis. Could be an adapter protein that facilitates and regulates interaction of KDR with effector proteins important to endothelial cell survival and proliferation; SH2 domain containing
SHC1 9606.ENSP00000401303 0.311042654028436 0.5229448491155047 rgb(255,255,255) SHC-transforming protein 1; Signaling adapter that couples activated growth factor receptors to signaling pathways. Participates in a signaling cascade initiated by activated KIT and KITLG/SCF. Isoform p46Shc and isoform p52Shc, once phosphorylated, couple activated receptor tyrosine kinases to Ras via the recruitment of the GRB2/SOS complex and are implicated in the cytoplasmic propagation of mitogenic signals. Isoform p46Shc and isoform p52Shc may thus function as initiators of the Ras signaling cascade in various non-neuronal systems. Isoform p66Shc does not mediate Ras activation, [...]
SHC2 9606.ENSP00000264554 0.2189099526066351 0.6746618106139439 rgb(255,255,255) SHC-transforming protein 2; Signaling adapter that couples activated growth factor receptors to signaling pathway in neurons. Involved in the signal transduction pathways of neurotrophin-activated Trk receptors in cortical neurons (By similarity); SH2 domain containing
SMAD2 9606.ENSP00000262160 0.6693364928909953 0.35343392299687826 rgb(255,255,255) Mothers against decapentaplegic homolog 2; Receptor-regulated SMAD (R-SMAD) that is an intracellular signal transducer and transcriptional modulator activated by TGF-beta (transforming growth factor) and activin type 1 receptor kinases. Binds the TRE element in the promoter region of many genes that are regulated by TGF-beta and, on formation of the SMAD2/SMAD4

complex, activates transcription. May act as a tumor suppressor in colorectal carcinoma. Positively regulates PDPK1 kinase activity by stimulating its dissociation from the 14-3-3 protein YWHAQ which acts as a negative regulator
SMAD3 9606.ENSP00000332973 0.7000473933649289 0.2569719042663892 rgb(255,255,255) Mothers against decapentaplegic homolog 3; Receptor-regulated SMAD (R-SMAD) that is an intracellular signal transducer and transcriptional modulator activated by TGF-beta (transforming growth factor) and activin type 1 receptor kinases. Binds the TRE element in the promoter region of many genes that are regulated by TGF-beta and, on formation of the SMAD3/SMAD4 complex, activates transcription. Also can form a SMAD3/SMAD4/JUN/FOS complex at the AP-1/SMAD site to regulate TGF-beta-mediated transcription. Has an inhibitory effect on wound healing probably by modulating both growth and mi [...]
SMAD4 9606.ENSP00000341551 0.6292417061611375 0.24479708636836628 rgb(255,255,255) Mothers against decapentaplegic homolog 4; In muscle physiology, plays a central role in the balance between atrophy and hypertrophy. When recruited by MSTN, promotes atrophy response via phosphorylated SMAD2/4. MSTN decrease causes SMAD4 release and subsequent recruitment by the BMP pathway to promote hypertrophy via phosphorylated SMAD1/5/8. Acts synergistically with SMAD1 and YY1 in bone morphogenetic protein (BMP)-mediated cardiac-specific gene expression. Binds to SMAD binding elements (SBEs) (5'-GTCT/AGAC-3') within BMP response element (BMPRE) of cardiac activating regions (By s [...]
SMAD6 9606.ENSP00000288840 0.6718957345971565 0.09682622268470344 rgb(255,255,255) Mothers against decapentaplegic homolog 6; Acts as a mediator of TGF-beta and BMP antiflammatory activity. Suppresses IL1R-TLR signaling through its direct interaction with PEL1, preventing NF-kappa-B activation, nuclear transport and NF-kappa-B-mediated expression of proinflammatory genes. May block the BMP-SMAD1 signaling pathway by competing with SMAD4 for receptor-activated SMAD1-binding. Binds to regulatory elements in target promoter regions
SMAD7 9606.ENSP00000262158 0.677867298578199 0.17455775234131113 rgb(255,255,255) Mothers against decapentaplegic homolog 7; Antagonist of signaling by TGF-beta (transforming growth factor) type 1 receptor superfamily members; has been shown to inhibit TGF-beta (Transforming growth factor) and activin signaling by associating with their receptors thus preventing SMAD2 access. Functions as an adapter to recruit SMURF2 to the TGF-beta receptor complex. Also acts by recruiting the PPP1R15A- PP1 complex to TGFBR1, which promotes its dephosphorylation. Positively regulates PDPK1 kinase activity by stimulating its dissociation from the 14-3-3 protein YWHAQ which acts as a [...]
SMURF1 9606.ENSP00000354621 0.46545023696682464 0.1305411030176899 rgb(255,255,255) E3 ubiquitin-protein ligase SMURF1; E3 ubiquitin-protein ligase that acts as a negative regulator of BMP signaling pathway. Mediates

ubiquitination and degradation of SMAD1 and SMAD5, 2 receptor-regulated SMADs specific for the BMP pathway. Promotes ubiquitination and subsequent proteasomal degradation of TRAF family members and RHOA. Promotes ubiquitination and subsequent proteasomal degradation of MAVS. Plays a role in dendrite formation by melanocytes
SMURF2 9606.ENSP00000262435 0.4262085308056872 0.14177939646201876 rgb(255,255,255) E3 ubiquitin-protein ligase SMURF2; E3 ubiquitin-protein ligase which accepts ubiquitin from an E2 ubiquitin-conjugating enzyme in the form of a thioester and then directly transfers the ubiquitin to targeted substrates. Interacts with SMAD1 and SMAD7 in order to trigger their ubiquitination and proteasome-dependent degradation. In addition, interaction with SMAD7 activates autocatalytic degradation, which is prevented by interaction with SCYE1. Forms a stable complex with the TGF-beta receptor-mediated phosphorylated SMAD2 and SMAD3. In this way, SMAD2 may recruit substrates, such as [...]
SRC 9606.ENSP00000362680 0.3946445497630332 0.45551508844953176 rgb(255,255,255) Proto-oncogene tyrosine-protein kinase Src; Non-receptor protein tyrosine kinase which is activated following engagement of many different classes of cellular receptors including immune response receptors, integrins and other adhesion receptors, receptor protein tyrosine kinases, G protein- coupled receptors as well as cytokine receptors. Participates in signaling pathways that control a diverse spectrum of biological activities including gene transcription, immune response, cell adhesion, cell cycle progression, apoptosis, migration, and transformation. Due to functional redundancy be [...]
STAT3 9606.ENSP00000264657 0.41000000000000003 0.35811654526534864 rgb(255,255,255) Signal transducer and activator of transcription 3; Signal transducer and transcription activator that mediates cellular responses to interleukins, KITLG/SCF, LEP and other growth factors. Once activated, recruits coactivators, such as NCOA1 or MED1, to the promoter region of the target gene. May mediate cellular responses to activated FGFR1, FGFR2, FGFR3 and FGFR4. Binds to the interleukin-6 (IL-6)-responsive elements identified in the promoters of various acute-phase protein genes. Activated by IL31 through IL31RA. Acts as a regulator of inflammatory response by regulating differenti [...]
TGFB1 9606.ENSP00000221930 0.6070616113744076 0.35998959417273674 rgb(255,0,0) Transforming growth factor beta-1; Multifunctional protein that controls proliferation, differentiation and other functions in many cell types. Many cells synthesize TGFB1 and have specific receptors for it. It positively and negatively regulates many other growth factors. It plays an important role in bone remodeling as it is a potent stimulator of osteoblastic bone formation, causing chemotaxis, proliferation and differentiation in committed osteoblasts (By similarity). Stimulates sustained production of collagen through the

activation of CREB3L1 by regulated intramembrane proteolysi [...]
TGFB2 9606.ENSP00000355896 0.7273459715639811
0.390894901144641 rgb(255,255,255) Transforming growth factor beta-2; TGF-beta 2 has suppressive effects on interleukin-2 dependent T-cell growth; Endogenous ligands
TGFB3 9606.ENSP00000238682 0.601090047393365
0.2963059313215401 rgb(255,255,255) Transforming growth factor beta-3; Involved in embryogenesis and cell differentiation; Belongs to the TGF-beta family
TGFBR1 9606.ENSP00000364133 0.5934123222748816
0.23543184183142557 rgb(239,164,117) TGF-beta receptor type-1; Transmembrane serine/threonine kinase forming with the TGF-beta type II serine/threonine kinase receptor, TGFBR2, the non-promiscuous receptor for the TGF-beta cytokines TGFB1, TGFB2 and TGFB3. Transduces the TGFB1, TGFB2 and TGFB3 signal from the cell surface to the cytoplasm and is thus regulating a plethora of physiological and pathological processes including cell cycle arrest in epithelial and hematopoietic cells, control of mesenchymal cell proliferation and differentiation, wound healing, extracellular matrix production, immunosuppression and carcinogen [...]
TGFBR2 9606.ENSP00000351905 0.5251658767772512
0.23168574401664932 rgb(239,144,117) Transforming growth factor beta receptor 2; TGF-beta receptor type-2; Transmembrane serine/threonine kinase forming with the TGF-beta type I serine/threonine kinase receptor, TGFBR1, the non- promiscuous receptor for the TGF-beta cytokines TGFB1, TGFB2 and TGFB3. Transduces the TGFB1, TGFB2 and TGFB3 signal from the cell surface to the cytoplasm and is thus regulating a plethora of physiological and pathological processes including cell cycle arrest in epithelial and hematopoietic cells, control of mesenchymal cell proliferation and differentiation, wound healing, extracellular matrix [...]
TGFBR3 9606.ENSP00000212355 0.8254502369668247
0.3244016649323621 rgb(255,255,255) Transforming growth factor beta receptor type 3; Binds to TGF-beta. Could be involved in capturing and retaining TGF-beta for presentation to the signaling receptors; Proteoglycans
TRAF6 9606.ENSP00000433623 0.38014218009478673
0.20827263267429763 rgb(255,255,255) TNF receptor-associated factor 6; E3 ubiquitin ligase that, together with UBE2N and UBE2V1, mediates the synthesis of 'Lys-63'-linked-polyubiquitin chains conjugated to proteins, such as IKBKG, IRAK1, AKT1 and AKT2. Also mediates ubiquitination of free/unanchored polyubiquitin chain that leads to MAP3K7 activation. Leads to the activation of NF-kappa-B and JUN. May be essential for the formation of functional osteoclasts. Seems to also play a role in dendritic cells (DCs) maturation and/or activation. Represses c- Myb-mediated transactivation, in B-lymphocytes. Adapter protein that see [...]
VEGFA 9606.ENSP00000478570 0.5430805687203791
0.5538501560874091 rgb(0,178,157) Vascular endothelial growth factor A; Growth factor active in

angiogenesis, vasculogenesis and endothelial cell growth. Induces endothelial cell proliferation, promotes cell migration, inhibits apoptosis and induces permeabilization of blood vessels. Binds to the FLT1/VEGFR1 and KDR/VEGFR2 receptors, heparan sulfate and heparin. NRP1/Neuropilin-1 binds isoforms VEGF-165 and VEGF-145. Isoform VEGF165B binds to KDR but does not activate downstream signaling pathways, does not activate angiogenesis and inhibits tumor growth. Binding to NRP1 receptor initiates a signaling pathway needed fo [...]
VEGFB 9606.ENSP00000311127 0.47739336492891 0.8497918834547347 rgb(255,255,255) Vascular endothelial growth factor B; Growth factor for endothelial cells. VEGF-B167 binds heparin and neuropilin-1 whereas the binding to neuropilin-1 of VEGF-B186 is regulated by proteolysis
VEGFC 9606.ENSP00000480043 0.4833649289099526 0.7214880332986473 rgb(255,255,255) Vascular endothelial growth factor C; Growth factor active in angiogenesis, and endothelial cell growth, stimulating their proliferation and migration and also has effects on the permeability of blood vessels. May function in angiogenesis of the venous and lymphatic vascular systems during embryogenesis, and also in the maintenance of differentiated lymphatic endothelium in adults. Binds and activates KDR/VEGFR2 and FLT4/VEGFR3 receptors
XIAP 9606.ENSP00000360242 0.5439336492890996 0.11742976066597295 rgb(255,255,255) E3 ubiquitin-protein ligase XIAP; Multi-functional protein which regulates not only caspases and apoptosis, but also modulates inflammatory signaling and immunity, copper homeostasis, mitogenic kinase signaling, cell proliferation, as well as cell invasion and metastasis. Acts as a direct caspase inhibitor. Directly bind to the active site pocket of CASP3 and CASP7 and obstructs substrate entry. Inactivates CASP9 by keeping it in a monomeric, inactive state. Acts as an E3 ubiquitin-protein ligase regulating NF-kappa-B signaling and the target proteins for its E3 ubiquitin-protein ligas [...]
